# Risks, Release and Concentrations of Engineered Nanomaterial in the Environment

**DOI:** 10.1038/s41598-018-19275-4

**Published:** 2018-01-25

**Authors:** Bernd Giese, Fred Klaessig, Barry Park, Ralf Kaegi, Michael Steinfeldt, Henning Wigger, Arnim von Gleich, Fadri Gottschalk

**Affiliations:** 1 University of Bremen, Faculty of Production Engineering, Department of Technology Design and Technology Development, Badgasteiner Str, 1 28359 Bremen, Germany; 2 University of Natural Resources and Life Sciences, Institute of Safety and Risk Sciences, Borkowskigasse 4, 1190 Vienna, Austria; 3 Pennsylvania Bio Nano Systems, Doylestown, Pennsylvania 18901 United States; 4 Center for Environmental Implications of Nanotechnology (UC CEIN), University of California Santa Barbara, Santa Barbara, California, 93106-5131 United States; 5 GBP Consulting Ltd, Purton, UK; 6 Eawag, Swiss Federal Institute of Aquatic Science and Technology, Überlandstrasse 133, 8600, Dübendorf, Switzerland; 7 Empa, Swiss Federal Laboratories for Materials Science and Technology, Lerchenfeldstrasse 5, CH-9014 St. Gallen, Switzerland; 8 ETSS AG, Engineering, technical and scientific services, CH-7558 Strada, Switzerland

## Abstract

For frequently used engineered nanomaterials (ENMs) CeO_2_-, SiO_2_-, and Ag, past, current, and future use and environmental release are investigated. Considering an extended period (1950 to 2050), we assess ENMs released through commercial activity as well as found in natural and technical settings. Temporal dynamics, including shifts in release due to ENM product application, stock (delayed use), and subsequent end-of-life product treatment were taken into account. We distinguish predicted concentrations originating in ENM use phase and those originating from end-of-life release. Furthermore, we compare Ag- and CeO_2_-ENM predictions with existing measurements. The correlations and limitations of the model, and the analytic validity of our approach are discussed in the context of massive use of assumptive model data and high uncertainty on the colloidal material captured by the measurements. Predictions for freshwater CeO_2_-ENMs range from 1 pg/l (2017) to a few hundred ng/l (2050). Relative to CeO_2_, the SiO_2_-ENMs estimates are approximately 1,000 times higher, and those for Ag-ENMs 10 times lower. For most environmental compartments, ENM pose relatively low risk; however, organisms residing near ENM ‘point sources’ (e.g., production plant outfalls and waste treatment plants), which are not considered in the present work, may be at increased risk.

## Introduction

Owing to unique qualities and manifold variation possibilities of engineered nanomaterials (ENMs), numerous products and processes involving ENMs have been developed during the past decades^1–4^. Medium–term forecasts predict a constant growth of ENM production^5^. Without much doubt, the volume and variety of ENMs released into the environment during manufacture, transport, use, and disposal will increase accordingly^6^. This trend demands a comprehensive analysis of present and future toxin exposure in order to prepare for potential preventive measures^1^. Recent indications on the toxicity of already established ENMs reveal that widespread use of a substance should not be mistaken for evidence that the substance does no harm^7^.

As producers engage in abrasion, burning, cleaning, or degradation of matrices containing ENMs, all environmental compartments are likely to be exposed to ENMs. To date, the investigation of toxicological and, in particular, ecotoxicological effects of ENMs has lagged behind the study of their technical qualities and the development of new applications^8^. Synthetic amorphous silicas (SiO_2_-ENMs) have been used since the middle of the last century and are produced in large amounts^9^. In recent years, leading producers of SiO_2_-ENMs have increased their production capacities, in particular for mass applications such as tires^10,11^. Nanosilver (Ag-ENM) has been used for medical purposes since the beginning of the last century^12^. Similar to SiO_2_-ENMs, Ag-ENM is quite versatile in its applications. It has antimicrobial properties and is a component of many consumer products^13^. Unlike SiO_2_-ENMs, however, Ag-ENM has been studied broadly^14,15^. Turning to CeO_2_-ENM, a large percentage is used for chemical-mechanical planarization (CMP), and CeO_2_-ENM has also been used in automotive catalytic converters (ACCs) and as a fuel additive^16,17^. Given such established and diffuse applications, an analysis of the environmental distribution and concentration of the applied nanoparticles is advisable.

Compared with the toxicological examination of ENMs, few data are available about their actual release^18–20^. In terms of release, Ag-ENM is again the most studied ENM so far^21–24^. From a regulatory point of view, data on ENM environmental release and exposure are required to estimate the associated risk. Regardless of whether new or established ENMs are examined, models of exposure and their combination with toxicological data can contribute to a prospective risk assessment according to the precautionary principle^25–27^. However, this only applies to the extent that the uncertainty of exposure and toxicology models can be managed and their results validated based on reliable model input data; such data are mostly missing in ENM contexts^18^. Our stochastic approach takes into account, when necessary, relatively large uncertainty ranges from all model input parameters in the form of probability distributions, thereby generating the largest possible model output spectrum. Such uncertainty computation includes virtually all environmental release parameters as well as all raw toxic data used, effectively ensuring a conservative risk calculation.

The preliminary exposure analysis used in the present study combines elements of a scientific model of the fate and dispersal of ENMs with elements of an economic model of ENM use. Both models have separate sources of uncertainty. In principle, empirical measurements are subject to uncertainties associated with the precision, accuracy, and test relevancy of a scientific model. In particular, the analytical limitations of measurements in discriminating between natural and engineered colloids are potentially insurmountable^23^. Hence, to date, the results of models have been difficult to validate. The economic model is a snapshot in time of a dynamic marketplace that responds to price, availability, regulations, labeling, customer preferences, and other factors related to competitive materials. However, there is considerable uncertainty in the economic model, resulting from antitrust, patents, proprietary interests, and competitive advantage. Material flow analysis substitutes release data for the revenue of a market model^28^. These models often rely on assumptions that are known to be incorrect, such as balanced budgets or efficient markets. In such cases, the known incorrect assumptions serve to highlight or isolate parameters that would otherwise be lost in economic “noise”^29,30^. For the purpose of this study, which examines the individual’s potential for nanomaterial exposure in the German economy, two arbitrary yet reasonable decisions were necessary. The first decision was to limit the discussion to manufactured materials, thereby excluding the consideration of incidental and natural nanomaterial exposures such as pollution. The second decision involved the selection of a mass transfer model. Although reviewed literature^31^ provided justifications for each decision, it was necessary to retain simplifying assumptions with regard to the broad question of the potential for adverse health effects arising from these calculated exposures. In particular, care should be taken in comparing risk quantifications based on non-validated calculations^18^ with highly controversial minimal or exposure levels that assume no effects^32^.

Against this critical epistemic background, all past and future investigations of release and exposure in the field of ENMs confront a number of fundamental practical challenges. Basic obstacles include missing data on production volumes of ENMs and on the share used in different products. Meanwhile, some well-known databases provide information about products containing ENMs, but, with the exception of the Nanowerk Database^33^, they focus on consumer products such as household articles, cosmetics, or textiles. Thus, important, mass-relevant products like tires are probably underrepresented.

Because market studies are expensive to procure, access to them is limited. Moreover, even if the producers provided accurate datasets to market research organizations, such studies can provide only the quantities being produced at a given time. Furthermore, different studies can arrive at very different sets of results, which suggests that producers “guesstimate” amounts being produced and used (cp. suppl. inform. on global production volumes in Holden, *et al*.)^28^. Hence, these organizations hide their real data, which, unfortunately, makes conducting research even more difficult. Existing studies on ENMs are no less accurate than are those on other material segments. Because ENMs are relatively new, little history is available on which to base effective judgments about present and future production quantities (cp. Table S1b and market projections in Sun, *et al*.)^34^.

**Figure 1:**
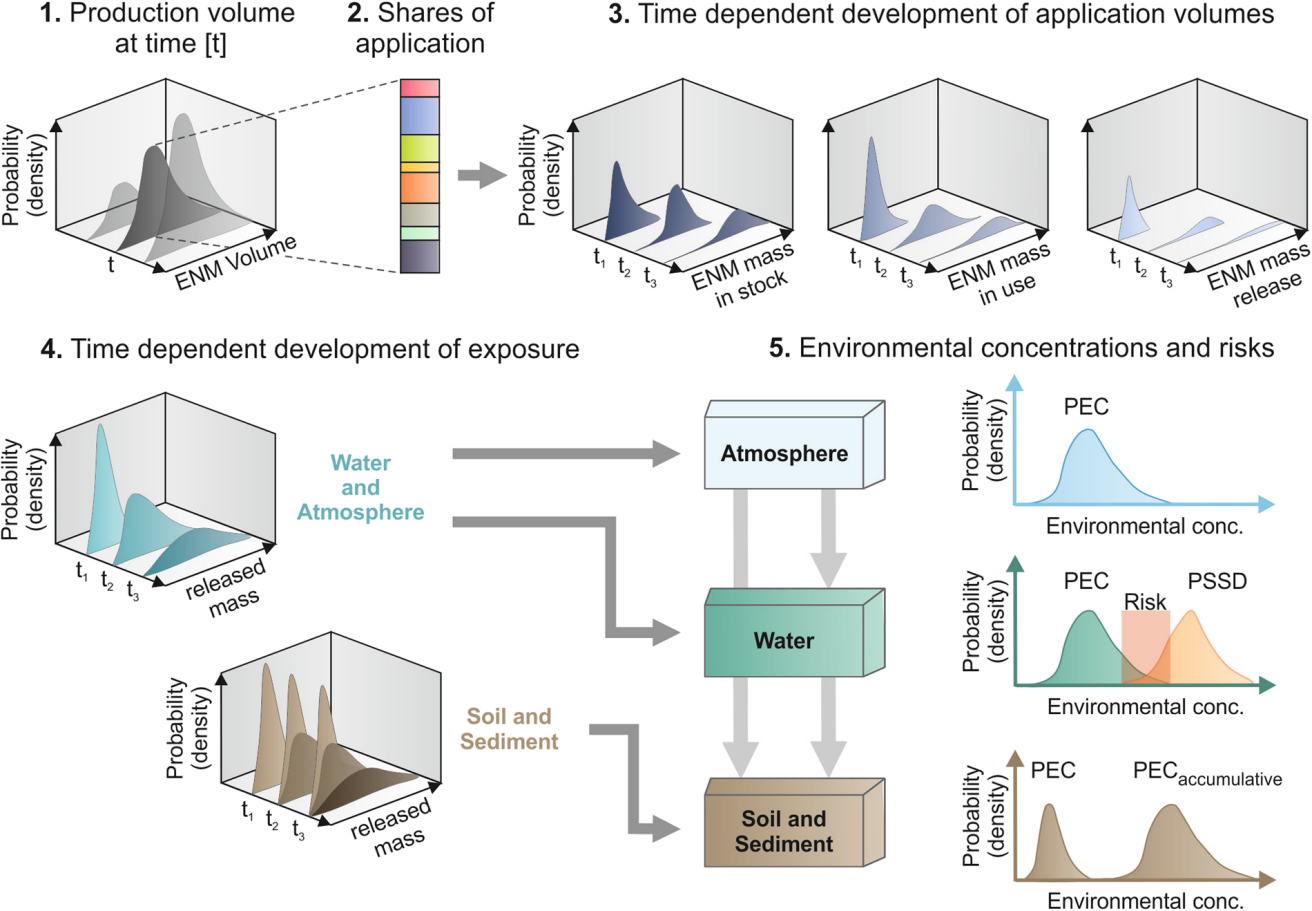
Data processing scheme for the calculation of a PEC and risk assessment. Each ENM was examined separately in the given order. After an initial estimation of the annual production volume of the respective ENM (1), according to the shares for different applications in processes and products, the mass fractions for these uses were calculated (2). Based on general trends for production volume, and taking into account factors for ENM release during its production, manufacture of ENM-containing products, use of ENMs and respective products, and end-of-life (EOL), the temporal distribution of ENM volumes for (i) stock, (ii) use, and (iii) release were determined stochastically (3). Information on release volumes enabled the computation of temporal probability distributions of ENM volumes in the atmosphere, water, and soil incl. sediment (4). To consider a large range of potential ENM concentrations for soil and sediment, we took into account two options for the persistence of ENM: on the one hand, an annual degradation of the released amount and, on the other hand, full ENM persistence and therefore an accumulation of annual releases that lead to considerably higher environmental concentrations of the respective ENM (PEC_accumulative_). Besides the relevant volume within the environmental compartments, flux between compartments has to be considered for the calculation of PEC (gray arrows). Comparison of PEC with probabilistic species sensitivity distribution (PSSD) curves for the three investigated ENMs enabled a risk evaluation for waterbodies.

Given the forecasted increasing use of CeO_2_-, SiO_2_- and Ag-ENMs in a variety of applications^5^, the goals for the present work were to model time-dependent past, current, and future use, release, and dispersal in natural and technical environments. Information on the annual global production volume of CeO_2_-, SiO_2_- und Ag-ENMs, as well as their annual trends, was researched based on a standardized questionnaire survey that involved producers, dealers, and research institutions in the field of nanomaterials. These investigations of use volumes were complemented by collecting and evaluating use data from available literature sources. Relevant product categories and the respective fractions of ENMs used for documented applications have been determined by a literature survey and an assessment of databases for nanomaterial applications. Product-life time-based transfer coefficients and factors describing the conversion of ENM into compounds without a nano-specific character enabled a time-dependent analysis of ENM transport and environmental release. The dynamic model used in this work takes into account the delay due to product storage and multi-year life cycles, as well as differences in transport and stability of ENMs. It allows an estimation of present and future mass-based release flows, dispersion into nature and the technosphere, and average environmental concentrations. The results may be used in future studies for covering spatial concentration heterogeneities by locally distributing the total German release. To enable an estimation of potential risks due to hazardous effects in soil and surface waters, a preliminary risk assessment was conducted by analyzing the overlap of predicted environmental concentration (PEC) and a probabilistic species sensitivity distribution for the respective ENMs^35^. A procedure for calculating PECs of the investigated ENMs and assessing potential risks is shown in Fig. 1.

The ENM exposure modelling done so far is mostly grounded on mass flow and multimedia fate and transport analysis, as well as their combinations^31^. Those models range from constant, annual release mass predictions on the continental or country level^36^ to dynamic, locally resolved computations with detailed ENM fate models integrating climatic and temporal variation^31^. We mainly focus on release and do not provide mechanistic fate modeling. ENM fate in natural and technical environments is covered as simplified mass transport reflecting erosion, sedimentation, dissolution, and other effects. However, this approach is far from proper mechanistic fate analysis, which is very challenging and is currently intensively researched and discussed^37–52^. Our modeling emerges from the initial stochastic probabilistic mass flow modeling^36^ performed on several occasions for different ENMs and with mostly constant release, temporal, and geographic conditions^53–58^. Dynamic stochastic modeling was first developed for unilateral flows of ENM transport in incineration plants^58^. Similar attempts to analyze environmental release variation over time for ENM analysis of general environmental exposure have been done recently based on other models^4,34,59,60^. Several further ENM modeling studies tracing materials through economy into nature have been conducted in various other labs^61–65^. We go a step further in release estimations at the regional (country) level by fully dynamizing the ENM life cycle and end-of-life (EOL) input from ENM product use and disposal, as well as accounting for data that suggest ENM dispersion inside the target system. Nevertheless, our intercompartmental transport only reflects annual mass transfer rates at the country level; it does not mechanistically include aggregation, advection, aerosol attachment, suspension, resuspension, and other ENM fate processes, as has successfully been performed on local scales^31^. Three stochastic models cover (i) ENM release and subsequent transport between giving and receiving compartments, (ii) ENMs in economic circulation (delayed release), and (iii) ENMs already out there in nature and technical sinks over a large time period of up to one century (in extremes from 1950 to 2050). For the first time, we attempted to distinguish predicted concentrations derived from ENM use (USE) phase release, and potential after-use (EOL) release. Finally, we will scrutinize our predictive ENM modeling outputs by cautiously and self-critically discussing their validity and plausibility and comparing our predictions to results of first initial measurements.

## Results

### ENM Production Volumes

Large variance in our survey and in the literature reflects extreme uncertainty regarding ENM production volumes in the nanomaterials community (Table 1). For SiO_2_-ENM, estimates in the survey vary between a global production of more than 100,000 and roughly 3 million tons per year. Figures from literature begin far below 100,000 tons per year^66^ and range up to 1.5 million tons^67^.

**Table 1:** Estimates on global engineered nanomaterial production volumes from a survey and literature. For a detailed presentation of data from literature, see Table S1b.

Source	SiO_2_-ENM in t/a	CeO_2_-ENM in t/a	Ag-ENM in t/a
Company A	>1,000,000	10,000–100,000	1–10
Company B	ca. 3,000,000		
Company C	>100,000	1,000–10,000	10,000–100,000
Company D			1,000–10,000
Institute A	>100,000	1,000–10,000	100–1,000
Literature	55–1,500,000	5.5–10,000	5.5–550

Compared with numbers in the literature^20,62,67^ survey data for CeO_2_-ENM reflect the upper limit of 10,000 tons per year, except for one of three estimates, which is higher by one order of magnitude.

For Ag-ENM, all data from the literature indicate a production volume below 1,000 tons per year^2,20,21,56,66,67^. Therefore, two of four estimates in our survey almost certainly represent extreme overestimates of the annual production and thus have been omitted for further modeling.

Estimates from our survey and data from literature were used to model a probability distribution of the global production volume. Regardless of their origin, all data from literature (Table S1b) and our own survey where integrated with equivalent weighting. Before computing a distribution, data from literature were scaled for 2015 conditions using trends for the annual produced amount (see next section).

### Trends for annual produced amount of ENM

On reviewing our survey results and a recent project report for the European Commission^5^, we derived a median annual growth of 5% (with 50% uncertainty/variability on each side) for the time period after 2000, which we incorporated in our modeling approach (Supplementary Table S2). For individual engineered nanomaterial applications before 2000, a median annual growth of 1% was chosen based on application trends for Ag-ENM for medical purposes^34^ (Supplementary Table S2).

### ENM Applications

Based on the evaluation of a combined investigation for applications of SiO_2_-, CeO_2_-, and Ag-ENMs, we identified product categories for each ENM.

Synthetic amorphous silica can be differentiated into pyrogenic or precipitated silica, silica gel, or sol; each has unique physical and chemical properties that may lead to different toxicological effects. Toxic effects and indications for inflammatory reactions are reported for pyrogenic (fumed) silica in cell culture assays^68–70^. For colloidal silica, only minimal overall toxicity was shown by Zhang, *et al*.^68^. Therefore, the different polymorphs of SiO_2_-ENMs are treated separately in the present work. According to a study by the European Centre for Ecotoxicology and Toxicology of Chemicals (ECETOC) on SiO_2_-ENMs^9^, pyrogenic silica is predominantly used in elastomers, polyester and epoxy resins, adhesives and sealants, and paints and coatings (Supplementary Table S3). Major applications of colloidal silica include the production of pulp and paper, refractories, and casting processes. Among numerous other applications, silica gel is primarily used as a food or tableting additive, as well as in paints, coatings, and plastics. The greatest proportion of precipitated silica is used for rubber products like tires and shoe soles^9,57^.

The investigation of product categories for CeO_2_-ENMs revealed a number of different applications (Supplementary Table S4). Due to its oxygen storage capacity, CeO_2_-ENMs are contained in the catalytic layer of ACCs and used as a fuel additive in diesel engines. Other applications include exterior coatings (e.g., UV-protective varnish for wood), integration in mixtures for CMP of glass and silicon wafers, and use in nickel–metal hydride (NiMH) batteries.

Although, for Ag-ENMs, absolute use volumes are low compared with SiO_2_- and CeO_2_-ENMs, Ag-ENMs are widely used. According to our investigation of its major product categories, it is used primarily in consumer electronics, in textiles, and for medical purposes (Supplementary Table S5).

### ENM Releases

For each individual product category, a release dynamic was assumed using a transfer coefficient (TC) as a simplified representation of the release during (a) production, formulation, and manufacturing processes (Supplementary Table S6); (b) a product’s use over its life span (Supplementary Tables S7–S9); and (c) end of life (Supplementary Tables S7–S9). TCs were mainly derived from data given in the literature. However, a complete list of the life cycle phases for a number of applications are not covered by existing publications on environmental release, such as EOL for use in SiO_2_-ENM investment casting or CeO_2_-ENM as a fuel additive (cp. Supplementary Tables 7 and 8, resp.). In such cases, TCs for EOL were obtained from literature focused on the respective processes or by primary research. There was not any information available regarding the use phase of some other applications. In these cases, assumptions were derived from environmental release categories (ERC) from the European Chemicals Agency (ECHA)^71^ or adopted from comparable releases of other materials (see footnotes of Supplementary Tables 7–9). For example, TCs for CeO_2_-ENMs used in chemical-mechanical-polishing (CMP) are based on ERC No. 12a, ‘Processing of Articles at Industrial Sites with Low Release’, and a report on recycling of rare earths^72^. Given the high number of different applications for the three ENMs investigated, a ‘patchwork’ approach in terms of TCs was inevitable due to the lack of available data. Corresponding uncertainties were taken into account by transferring TCs into probability distributions.

Overall, emissions in the first phase of the life cycle during production, formulation, and manufacturing processes are low and mainly associated with solid waste disposal, followed by emissions to waste water and a minor fraction to air. According to factors given in literature, release can be expected to not exceed 1% of the total ENM mass contained in most products, except for formulations of suspensions, where a release of 1–5% must be considered (Supplementary Table S6).

TCs for release during product life have been arranged separately for each product category. In accordance with the differentiation for individual types of synthetic amorphous silica, TCs of product applications have been treated separately (see Supplementary Table S7). Relevant release sources for *pyrogenic silica* (due to share of product application and TC) are (a) non-silicones like pharmaceuticals, cosmetics, toner, batteries, or food with a major release to waste water and a release-enhancing product lifespan of only one year; and (b) silicones-elastomers and paints or coatings with a major release to soils. Although their release is distributed over a long product lifespan of approximately ten years, a high fraction of early release is assumed for paints and coatings.

For *colloidal silica*, excepting clarification processes of wine, beer, and fruit juice, which account for only about one-tenth of colloidal silica applications, we assume only very low release.

Important applications of *gel silica* are (a) pharmaceutical, food, and dentifrices; and (b) textiles with a high TC to waste water. However, for textiles, the release is stretched over an assumed medium product lifespan of five years. For absorbents and desiccants containing gel silica, a high release was assumed for all compartments, but they represent only a minor share with regard to product applications. Release-relevant applications of *precipitated silica* are (a) rubber products like tires and shoe soles, with a high TC to soils due to dust from streets; and (b) toothpaste and cosmetics with a major release to waste water.

Due to preliminary evidence for differential toxicological impacts of the different types of silica, it seems necessary to include, in a discussion on potential risks, an estimate of the contributions of silica types to overall release. Wastewater is an important compartment for release of silica during the use phase. According to its annual release masses into waste water, with 62% precipitated silica shows the highest mean annual contribution. In the outcome of our modeling approach, precipitated silica is followed by gel silica (22%), pyrogenic silica (12%) and colloidal silica (4%).

A relevant source of CeO_2_-ENM emissions may result from (a) improper disposal of small NiMH batteries or (b) (in a worst-case scenario for fabrication conditions) from CMP during manufacturing of optical or electronical equipment (see Supplementary Table S8). Although absolute numbers of TCs are lower, release from ACCs and diesel fuel additives represents a diffuse source whose potential for minimizing ENM emissions is smaller than for CMP or batteries, largely as a result of being contained within a diesel particulate filter or trap.

According to our investigations, most released nanosilver is derived from textiles during washing processes (see Supplementary Table S9). Cleaning agents and cosmetics are also relevant sources of nanosilver, with a release path via waste water. Medical products, as the oldest application for nanosilver, contribute to release as well, with emphasis on emission to waste water.

**Figure 2:**
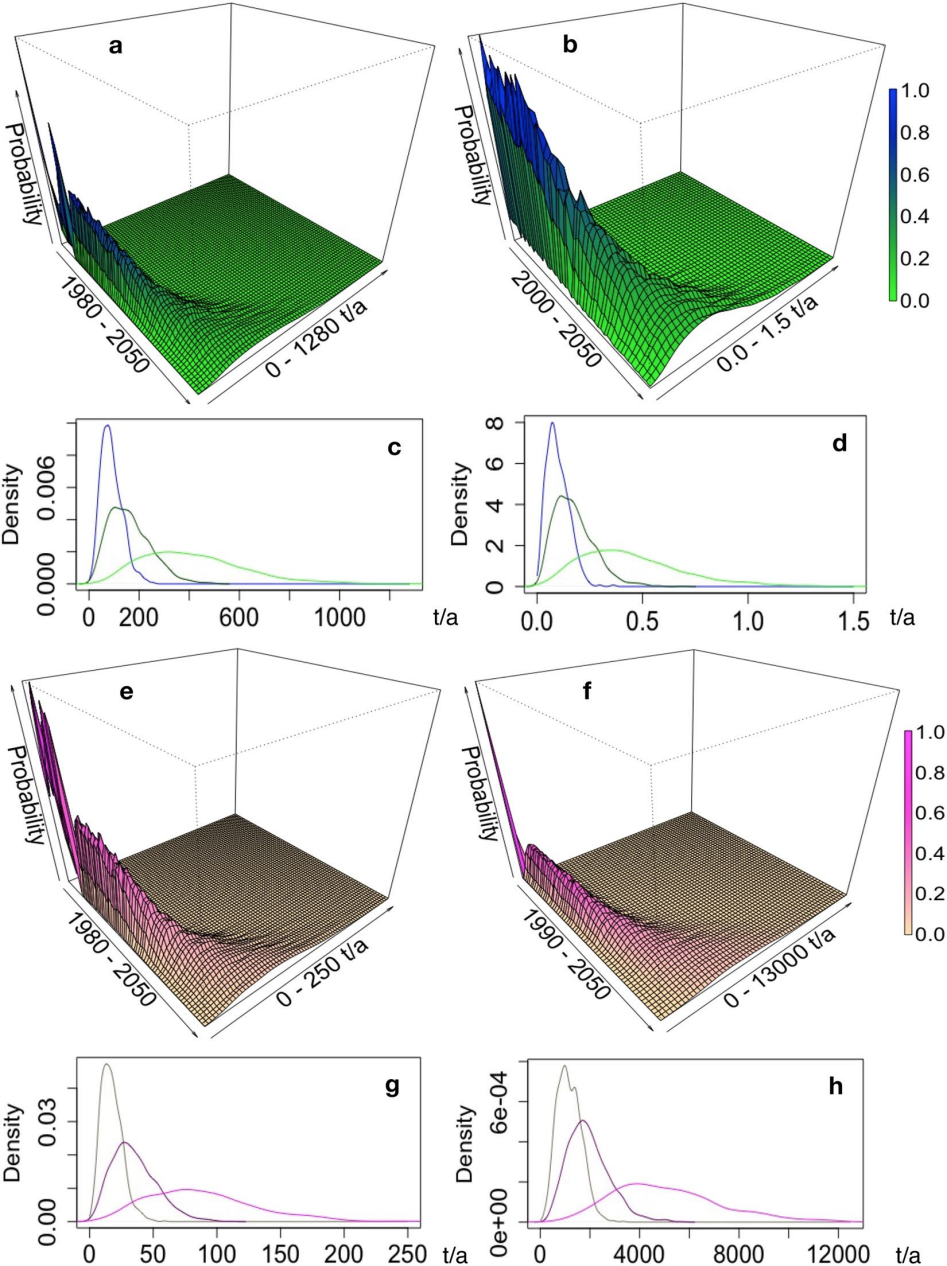
CeO_2_-ENM released into natural (waters, soils, air) and technical compartments (landfills, recycling, waste incineration, and sewage treatment). The former release is shown in the Figures (**a**–**d**), the latter in the Figures (**e**–**h**). The left side (**a**,**c**,**e**,**g**) shows ENM released during product uses. The right side (**b**,**d**,**f**,**h**) represents release during ENM products’ end-of-life phase. The corresponding relative density curves (**c** taken from **a**, **d** taken from **b**, **g** taken from **e**, **h** taken from **f**) represent, from left to right, outputs for the years 2017, 2030, and 2050. Shown are the whole ranges (range with some probability) of the stochastic modeling. Scenarios that theoretically run all ENM applications simultaneously at the lowest, or, in another scenario, at the highest ENM production, use and environmental release are not considered here.

**Figure 3:**
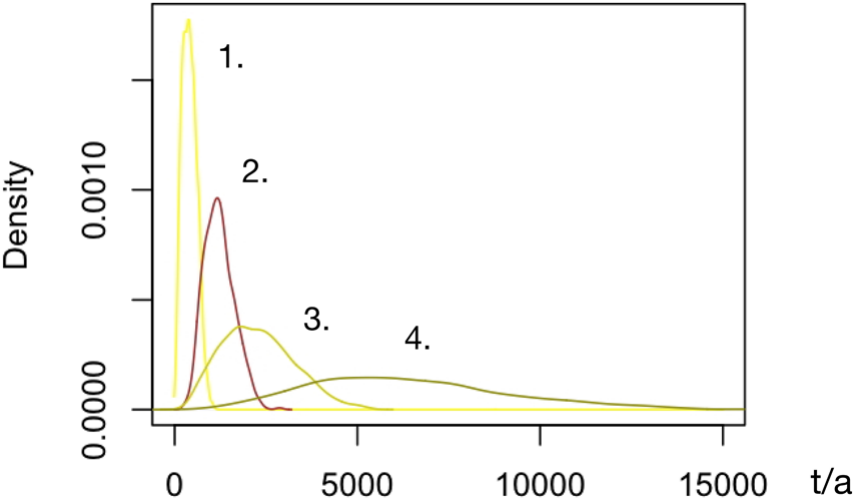
SiO_2_-ENM contributions in t/a (*x* axis) to use release into wastewater in 2017 (most probable range). From left (mean contribution to total release): 1. colloidal silica (4%), 2. pyrogenic silica (12%), 3. gel silica (22%), and 4. precipitated silica (62%).

**Figure 4:**
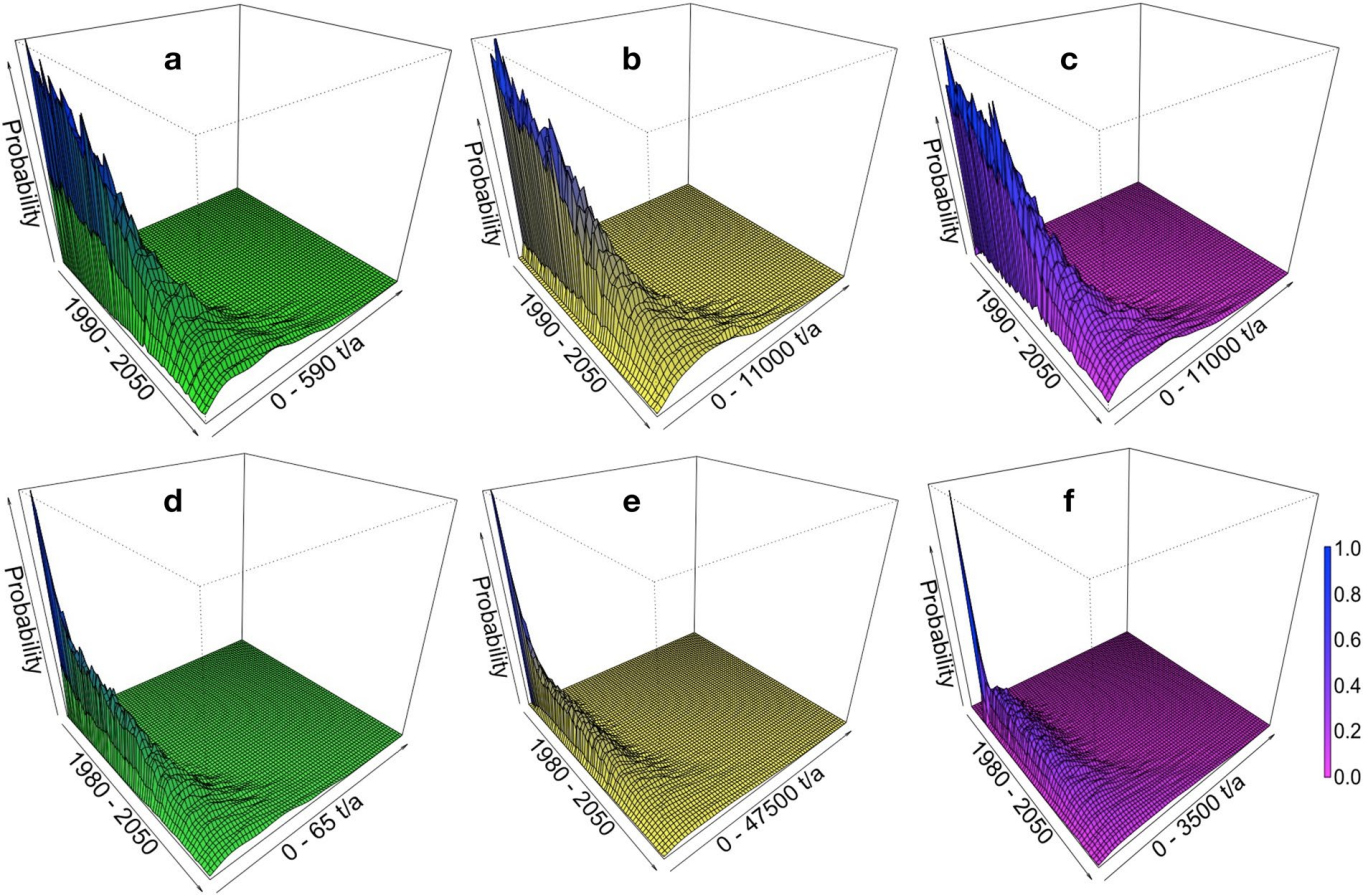
Predicted ENM mass (most probable range) between 1990 and 2050 (1980 and 2050) from two exemplary and relevant CeO_2_-ENM applications. From left: ENM release mass to nature (**a** and **d**), ENM mass (**b** and **e**) in circulation (release delay during product use), and ENM mass (**c** and **f**) deposited or eliminated in technical treatment (landfilling, recycling, waste incineration, and sewage treatment). Above (**a**–**c**), chemical-mechanical polishing (CMP) application; below (**d**–**f**), use for ACCs (automotive catalytic converters).

As seen in Fig. 2, for CeO_2_, release of ENMs to nature is dominated by release during the use cycle; however, the overall material transfer will be dominated by the flow into the technical compartments at the EOL phase. Assuming that we can reliably control most uncertainty and natural and economic variability in its main input parameters, the model (Fig. 2) effectively predicts the expected total mass transport and release spectrum. However, the prognostic precision disappears towards the middle of the century, where no clear tendency appears in the results. Based on the current data, the EOL release to nature is insignificant and smaller by a factor of 1,000 than the release from ENM product use. Table 2 confirms that, for all materials and time periods studied, release into nature is much lower than EOL mass transfers into technical sinks. Only silica reveals a significant discharge to nature, smaller only by a factor of approximately 2 than the mass ending up in technical sinks. This is explained by a large and diverse nano-silica application spectrum, with some applications leading to direct release into natural compartments during their use, like paints and coatings or rubber products with abrasive uses (e.g., tires or shoe soles). As seen in Fig. 3, the different nano-silica types vary considerably regarding their use-based release properties. Precipitated nano-silica contributes more than half of the waste water–based aquatic discharge. As we see in Fig. 4 and Table 3, for the CeO_2_ case, the different ENM applications considerably differ regarding their use life cycle and release to nature, as well as regarding their disposal and other EOL flows. From the CMP application, approximately 5% of mass may end up in natural environments, whereas, from ACCs, around 1% is released to nature. We learn that specific consideration of each application and its life cycle duration is necessary to significantly improve precision in this kind of release and subsequent exposure and risk assessment. A survey of current mean mass movements is given in the Supplementary Information (Figs S2–S4).

**Table 2:** Ag-, CeO_2_-, and SiO_2_-ENM release in t/a to nature (waters, soils, air) and the technosphere (landfills, recycling, waste incineration, and sewage treatment). Shown are rounded and simplified modal values (most frequent Monte Carlo outputs) for use and end of life (EOL) release. For CeO_2_ results, see also Fig. 2 and, for specific flow charts, Figs S2–S4 in the Supplementary Information.

		**2017**		**2030**		**2050**	
		Use	EOL	Use	EOL	Use	EOL
Ag-ENM	*Nature*	<5	≪1	≈5	≪1	≈15	≪1
	*Technosphere*	<2	≈10	<5	≈15	<10	≈30
CeO_2_-ENM	*Nature*	≈75	<1	≈ 100	<1	≈300	<1
	*Technosphere*	≈13	≈1000	≈30	≈1700	≈75	≈4000
SiO_2_-ENM	*Nature*	≈17000	<5	≈30000	≈ 5	≈73000	≈15
	*Technosphere*	≈5250	≈36000	≈12000	≈55000	≈30000	≈145000

**Table 3:** Predicted annual mass (most probable range) in tons (see corresponding Fig. 4) of CeO_2_-engineered nanomaterial from use in single applications. CMP stands for chemical-mechanical polishing, ACC for automotive catalytic converters. In bold: mass in circulation (release delay during product use); italic: release mass to nature; and bold italic: ENM mass deposited or eliminated in technical treatment (landfilling, recycling, waste incineration, and sewage treatment).

Single ENM applications		**2017**			**2030**			**2050**		
		min	mode	max	min	mode	max	min	mode	max
**In circulation**	**CMP**	**<3**	mean* ≈600	**≈3000**	**<5**	**≈1000**	**≈5000**	**<40**	**≈2000**	**≈11000**
	**ACC**	**<10**	**≈3000**	**≈10000**	**<20**	**mean ≈5000**	**≈20000**	**<200**	**mean ≈13000**	**50000**
*Release to nature*	*CMP*	*≈0*	*mean ≈30*	*≈120*	*<1*	*mean ≈60*	*≈250*	*<1*	*mean ≈150*	*≈600*
	*ACC*	*≈0*	*<2*	*≈10*	*≈0*	*≈2*	*≈20*	*<1*	*mean ≈15*	*≈70*
***Release to sinks***	***CMP***	***<5***	***mean ≈500***	***≈2500***	***<5***	***≈450***	***≈4000***	***≈5***	***mean ≈2850***	***≈11000***
	***ACC***	***<1***	***≈250***	***≈900***	***<1***	***mean ≈400***	***≈1500***	***<5***	***mean ≈1100***	***≈3500***

*If no clear tendency there for mode computation.

### ENM Concentrations

**Figure 5:**
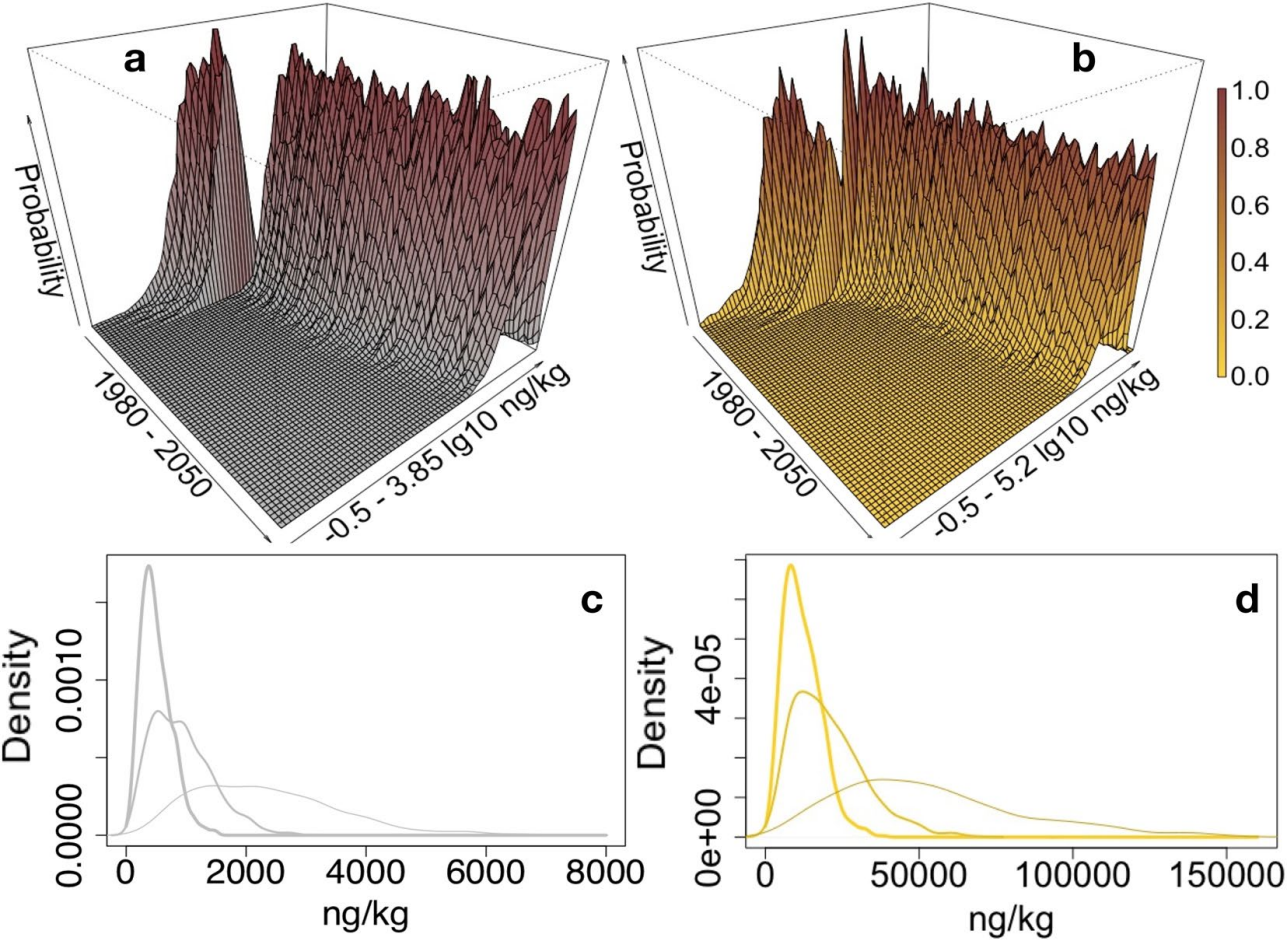
Temporal dynamics of CeO_2_-ENM PEC (most probable range) for agricultural soils (left side, scenario of immediate ENM degradation after one year (**a** and **c**); right side (**b** and **d**), persistent scenario of no ENM degradation or elimination at all after being released into soils). The density curves refer in each scenario (**c** out of **a**, and **d** out of **b**) to the results for 2017, 2030, and 2050 (from left).

**Figure 6:**
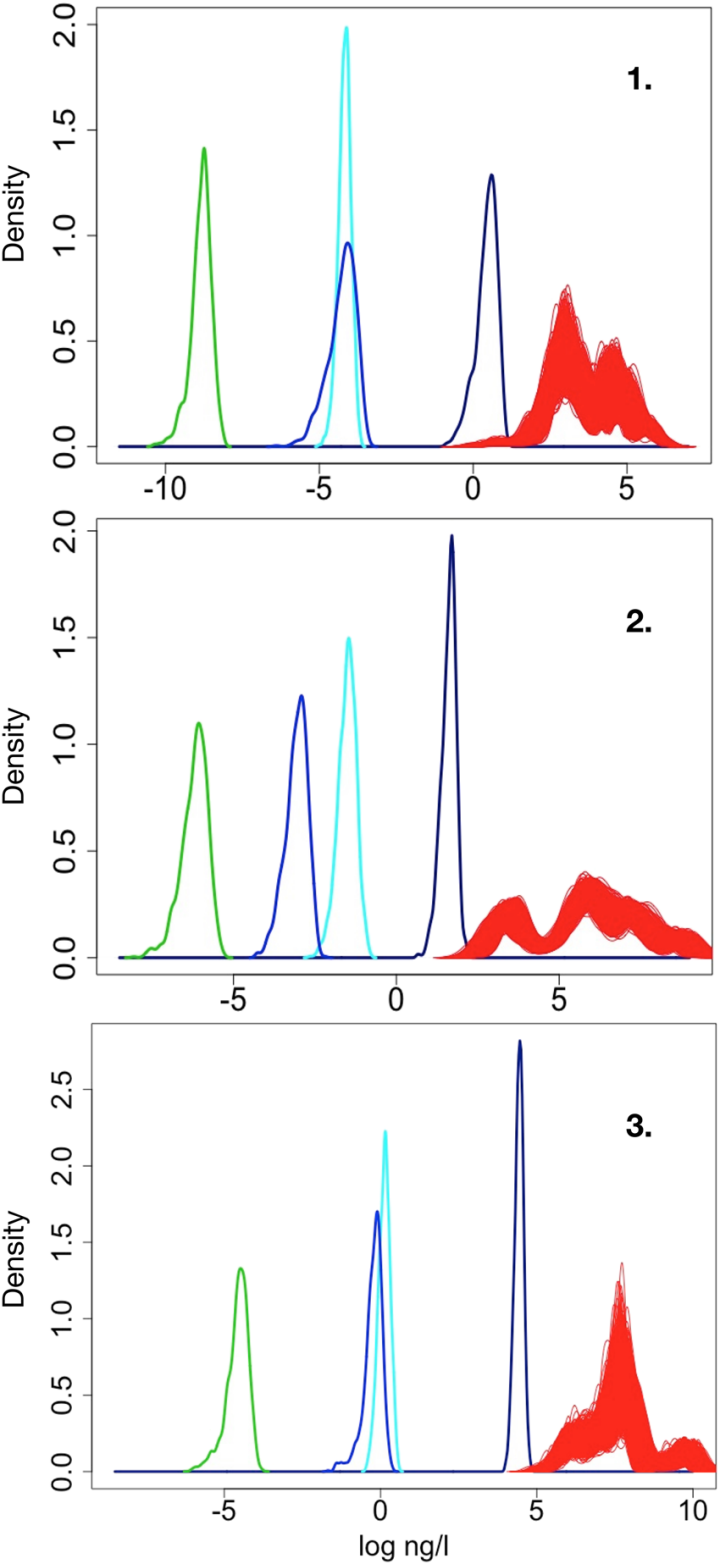
Water risk evaluations (1) for Ag-, (2) CeO_2_-, and (3) SiO_2_-ENMs by comparing PECs (most probable range) of 2050 and 1,000 PSSD curves (in red). The PEC curves (form left to right) represent marine water (green) loaded only from releases associated with end of life (EOL) of ENMs, in blue marine water loaded from ENM product uses, and EOL releases followed by the equivalent freshwater loadings from EOL (light blue) and total (use and EOL) release (dark blue).

The model output reveals (as presented in Table 4 and Supplementary Tables S15–S17, Figs 5 and 6) that it is impossible to establish any deterministic concentration prediction for ENMs. However, it is possible to show some probable tendencies and to explore the probable and possible range of concentrations. The full dynamic model output spectrum in one year and over the considered time periods (1950 to 2050) spans in extremis several orders of magnitude. The lowest aquatic ENM concentrations were predicted for silver and ranged (full spectrum) for fresh water in 2017 from a few pg/l to some ng/l. For 2030 and 2050, these predictions are, roughly speaking, about two- and sixfold higher, with most probable 2050 concentrations ranging from approximately 100 pg/l to 10 ng/l. The PECs for marine waters reveal, without exception, marginal values below pg/l levels and, in 2050, at the highest extremes, up to 1 pg/l. The fresh sediment concentrations range from a few pg/kg in 2017 (fast ENM degradation after one-year residence) to a few mg/kg in 2050 (no ENM degradation in nature). The marine sediment outputs are up to tenfold smaller and probably do not achieve mg/kg levels by 2050. In terrestrial contexts, the same time and scenario comparisons yield a range for agricultural soils between 30 pg/kg (min., 2017) up to 10 µg/kg soil (max., 2050). Similar outputs were seen for natural and urban soils. For sludge-treated soil areas, our predictions were about a factor of 30–40 higher than for agricultural soils. Equivalent time range predictions in the atmosphere (min. 2017 to max. 2050) span from low or below pg/m^3^ to a few ng/m^3^.

**Table 4:** PECs for 2017 for SiO_2_−, CeO_2_−, and Ag-ENMs. Shown are the most frequently modeled (mode) values; modes that only reflect concentrations from end of life (EOL) releases; ranges (range with some probability) of stochastic modeling that do not necessarily force all applications simultaneously running at the lowest, or, in another scenario, at the highest production; and use and environmental release levels. Such simultaneous extreme scenarios are provided in the absolute min-max borders on the left and right sides. The max border shows extreme high-end spectrum values, which are possible but not very likely, with the assumption that the entirety of ENM import and production (all applications) are simultaneously running at maximum levels with subsequent high ENM use and release. We have rounded the model output to two places after the decimal point. Nevertheless, the results included after the decimal point values should not suggest any accuracy of individual values taken from the analysis of probability distributions. This table rather shows the expected full spectrum with modeled trends.

	Unit	Min	Mode	(Mode EOL)	Range	Max
**SiO**_**2**_ **-ENM**						
*Sewage treatment effluent*	µg/L	0.01	44.37	0.01	9.04–821.53	1,557.61
*Surface water (fresh water)*	µg/L	0.00	5.34	0.00	1.80–11.63	25.55
*Sea water*	ng/L	0.00	0.12	0.00	0.01–0.39	1.11
Sediments (fresh water)*	mg/kg	0.00	32.12	0.00	0.15–131.13	302.82
Sediments (fresh water)**	mg/kg	0.00	920.81	0.04	4.63–3,849.29	8,681.59
Agricultural soils*	µg/kg	0.00	62.82	0.01	25.61–127.75	258.17
Agricultural soils**	µg/kg	0.08	1,698.93	0.33	945.72–2,622.65	6,981.61
Sludge treated soils*	µg/kg	0.11	3,085.15	0.42	1,125.36–6,508.83	11,603.86
Sludge treated soils**	µg/kg	2.85	78,275.11	9.41	32,021.29–177,840.16	294,408.31
*Air*	ng/m^3^	0.00	13.92	0.10	1.89–51.36	79.43
**CeO**_**2**_ **-ENM**						
*Sewage treatment effluent*	ng/L	0.03	199.92	0.26	20.30–888.75	1,867.90
*Surface water (fresh water)*	ng/L	0.00	7.01	0.01	0.51–25.59	61.74
*Sea water*	ng/L	0.00	0.00	0.00	0.00–0.001	0.003
Sediments (fresh water)*	µg/kg	0.00	49.03	0.02	0.34–253.49	611.95
Sediments (fresh water)**	µg/kg	0.04	901.12	0.54	4.40–8,328.88	13,496.61
Agricultural soils*	ng/kg	0.10	377.41	0.30	43.67–1,491.87	2,943.37
Agricultural soils**	ng/kg	2.10	7,963.94	6.24	1,314.06–36,210.88	62,109.92
Sludge treated soils*	ng/kg	0.97	4,450.17	6.65	528.57–19,011.99	35,069.36
Sludge treated soils**	ng/kg	25.96	118,515.88	138.04	11,212.11–560,423.28	933,959.89
*Air*	ng/m^3^	0.00	1.23	0.00	0.12–5.53	12.10
**Ag-ENM**						
*Sewage treatment effluent*	ng/L	0.06	18.89	0.00	1.24–103.79	151.25
*Surface water (fresh water)*	ng/L	0.00	0.38	0.00	0.03–2.79	4.17
*Sea water*	ng/L	0.00	0.00	0.00	0.00–0.000	0.000
Sediments (fresh water)*	µg/kg	0.00	1.85	0.00	0.02–33.67	47.02
Sediments (fresh water)**	µg/kg	0.00	29.55	0.00	0.19–470.65	749.35
Agricultural soils*	ng/kg	0.03	7.76	0.00	0.24–67.73	80.30
Agricultural soils**	ng/kg	0.50	147.13	0.02	9.40–792.23	1,522.66
Sludge treated soils*	ng/kg	1.13	257.18	0.01	20.08–1,661.16	2,294.05
Sludge treated soils**	ng/kg	22.66	5,141.25	0.25	464.15–24,995.35	45,859.26
*Air*	ng/m^3^	0.00	0.06	0.00	0.00–0.49	0.58

*100% degradation scenario after one year.

**100% persistent ENM (no-degradation scenario).

The 2017 CeO_2_-ENM full-spectrum concentrations predicted for fresh waters range from, minimally, 1 pg/l in 2017 to, maximally, a few hundred ng/l in 2050. The most probable current concentrations range approximately from 1 ng/l to 30 ng/l. Probable values for 2030 and 2050 are about 2–3 and 4–6 times higher, respectively. The maximal PECs for marine waters achieve, at the most, about 10 pg/l in 2050. The fresh sediment predictions span from fast ENM degradation values of a few ng/kg in 2017 to some mg/kg for the no-degradation scenario. The sea sediment equivalents are, on average and roughly, a factor 6–10 lower. The time and min-max scenario comparisons between 2017 and 2050 reveal, for agricultural soil PECs, a range from 100 pg/kg up to 400 µg/kg. Comparable outputs were again computed for natural and urban soils. The sludge-treated soil predictions were roughly a factor of 10–20 higher than those for agricultural soils. Our air PECs (min. 2017 to max. 2050) range approximately from a few pg/m^3^ to about 50 ng/m^3^.

The highest predictions concern the SiO_2_-ENM concentrations. The fresh water extreme PECs range, in 2017, from pg/l levels to around 30 µg/l. The current most probable concentrations lie around 1–10 µg/l. In 2030 and 2050, these values are, again very roughly, 2 and 4–6 times higher, respectively. The marine water high-end extreme PECs are, in 2050, at the most around a few ng/l. Amorphous SiO_2_-ENMs have a low water solubility^9^. It is therefore expected that the vast majority of released SiO_2_-ENMs settle in soil and sediment. The ENM distributions obtained by our model for marine and fresh water represent the whole mass of the respective ENMs, including dissolved and particulate ENMs. The concentrations in fresh sediment span from about hundred ng/kg in 2017 (fast ENM degradation) to low g/kg levels in 2050 for the no-degradation scenario. The sediment results for marine contexts are, again, in most cases smaller by a factor of 6 or 7, probably achieving, at the most, a few g/kg in 2050. The time and scenario comparisons for agricultural soils show values lying between 3 ng/kg (min. 2017) up to 30 g/kg soil (max. 2050). As seen before, the natural and urban PECs were similar. The sludge-treated soil results were in most cases about 30–40 times higher than the agricultural equivalents. The full-spectrum time and min-max range predictions for atmospheric PECs (2017–2050) span from the before-computed low, below the pg/m^3^ level, to some hundred ng/m^3^.

### ENM Risks

In Fig. 6 and Table 5, the risk probability plots for waters are shown. Those risks are predicted by contrasting our PECs for 2017, 2030, and 2050 to our probabilistic species sensitivity distribution (PSSD) modeling for the target materials. The risk computations involve concentrations in: (from right in Fig. 6) (i) freshwaters potentially contaminated by ENM product use release, combined with release from ENM products’ EOL treatment; (ii) fresh water contaminated only by EOL-based release; (iii) marine water contaminated by use and EOL release; and iv) marine water contaminated by EOL release alone. An overlapping of those concentrations with the toxic limit curves reveals possible risks for ecosystems. The quantified risk percentages (Table 5) combine the probability of critical PECs, defined as values higher than the lowest PSSD, with the probability of the PSSD curve being below the maximal PEC. For the first half of this century, our results suggest, at most, low risks for natural waters loaded with the ENMs studied. EOL-based exposure and seawater concentrations seem, with some reservations as discussed at the end of this section and applying to all risk statements made here, so far harmless for all water organisms. For CeO_2_-ENMs, risk is almost nonexistent. The Ag-ENM results for freshwater (use- and EOL-based ENM load) show risk values up to 1.4%, suggesting the possibility (at all time points studied) of exposure to a small fraction of water organisms. The same applies to SiO_2_-ENMs, where, at least for 2050, significant exposure cannot be excluded. With the exception of Ag and SiO_2_, the results show extremely low to no risk. However, a fly in the ointment is that, as seen in Fig. 6, by 2050 almost all Ag- and SiO_2_-ENM PECs for fresh water are toxic for at least a small fraction of organisms. Another reason for caution is that our PEC simulations do not address short-term local extremes, which could lead to much higher loads. In extreme cases, Gottschalk, *et al*.^54^ found, for geographic and temporal variation influencing Swiss rivers, increase in ENM loads by factors up to the four- and five-digit range. Taking such extreme factors to hypothetically increase our freshwater total PECs would fully move the concentration curves of all target substances into the PSSD concentration levels (Fig. 6) resulting in all organisms being at risk.

**Table 5:** Risk results for Ag-, CeO_2_-, and SiO_2_-ENM in waters (see also Fig. 6). Risks are predicted by comparing PEC (most probable range) of 2017, 2030, and 2050 to PSSD outputs. The values represent fresh water loaded from ENM use and EOL releases, as well as fresh water loaded only from EOL release and marine water loaded from use and EOL, as well as only EOL, release. The percentages combine the probability of critical PECs (PEC values higher than the minimal PSSD) with the one of critical PSSDs (PSSDs smaller than the highest PEC).

Time	2017	2030	2050	2017–2050	2017–2050
Load	*Freshwaters with total ENM load*			End of life (EOL) phase loaded Freshwater	Seawaters with total ENM load
Ag-ENM	0.1%	0.7%	1.4%	0%	0%
CeO_2_-ENM	0%	0%	≈0%	0%	0%
SiO_2_-ENM	0%	0%	<0.1%	0%	0%

### Model results versus measurements

The validation of modeled PECs is currently hampered by lack of reliable data on environmental concentration of the ENMs. Nevertheless, recently concluded monitoring projects^73,74^ provided initial data on the occurrence of nanoscale CeO_2_ and Ag particles in effluent waters of wastewater treatment plants and in surface waters (Table 6). In these studies, samples were collected from 25 measurement points within the Bavarian survey network of watercourses. Amongst others, the authors of these reports used asymmetric flow field fractionation (AF4) in combination with (single particle) inductively coupled plasma mass spectrometry (ICP-MS)^73^ to detect and quantify nanoscale Ce- and Ag-particles. The target particles were detected in both, industrial waste waters and river waters. The reported concentrations have to be interpreted as maximum values as they refer to the total nanoscale particles detected in the respective samples and include natural, incidental and engineered NP. Nevertheless, reported concentrations of selected nanoscale particles are in good agreement with the respective modeled concentrations. The maximum value of the measured concentrations for nanoscale Ce particles is slightly higher than the respective modeled concentration of CeO_2_ nanoparticles in wastewater treatment plant effluents. For the Ag-NP, maximum measured concentrations are higher by about one order of magnitude compared to the modeled concentrations. Considering that very high effluent concentrations are probably caused by industrial wastewater treatment plants (and such spatial heterogeneities are not covered by the model) and that the model only includes engineered NP, the agreement between the modeled and the measured concentrations is surprisingly good. In the case of non-diluted waters, concentrations of silver – NP in our generic wastewater treatment plant effluents are in the same order of magnitude (ng/L) as the nanoscale silver reported from industrial wastewaters.

**Table 6:** Comparison for an early validity discussion of our PECs and nanoparticle concentration measurements, reported elsewhere^73^. We show full ranges with mode or mean values.

Compartment		*Modelled ENM*			*Measured nanoparticles*	
		Unit	Mode	Range	Mean	Range
CeO_2_-ENM	Sewage treatment effluent*	ng/L	199.90	0.03–1868.00	na	0.00–3900**
	*Surface water (fresh water)*	ng/L	7.00	0.00–61.70	5.10	0.00–13.80
Ag-ENM	Sewage treatment effluent*	ng/L	18.90	0.10–151.20	na	0.00–1300.00
	*Surface water (fresh water)*	ng/L	0.40	0.00–4.20	0.74	0.00–6.20

*Very vague comparison based on measurements for industrial waste water.

**Ce (non cerium dioxid) particles.

na: not available.

For CeO_2_, Park, *et al*.^17^ measured up to 0.5 ng/m^3^ near a busy road often frequented by Envirox-equipped busses. The authors also predicted, by assuming no sedimentation, 20–80 ng/m^3^ for a busy street canyon and 0.3–1 µg/g of soil 96 m from a road. These soil PECs are in the same range and about a factor of 10 higher compared with our lower values for presumably less exposed agricultural soils: 0.06 µg/g of soil (persistent scenario). Our most probable air PEC range of 0.1–5.5 ng/m^3^ is in the same order of magnitude as the concentrations reported by the Park *et al*.^17^ and correlate fairly well with their predictions for a busy street canyon. Furthermore, Erdakos, *et al*.^75^ confirmed such concentrations, predicting up to 20 ng/m^3^ for the United States.

## Discussion

The release and persistence of ENM into the environment was first detected a decade ago^76^. The investigation of production volumes of ENMs is of particular importance for the outcome of subsequent steps in exposure modeling. Our survey yielded numbers in the upper range of recent estimates from literature. However, there are indeed some indications for an increased production of ENMs. In the case of silica, leading manufacturers announced an increased productive capacity in recent press releases^10,11^. For CeO_2_-ENMs, we calculated the annual amount of ENMs needed for products and processes independent of estimates on production volumes: with a sum of approximately 22,000 t/a, the result of our demand-based calculation confirms the outcome of our survey on annual production volume. In the case of nanosilver, the large variance of volumes in our survey reveals a certain amount of ignorance regarding production volumes among the experts contacted in our survey. We expect an annual production volume below 1,000 t for nanosilver.

As important diffuse sources for the release of ENMs with no possibility for removal or separation of nanomaterials (e.g., in sewage treatment plants), we identified CeO_2_ in automotive applications and SiO_2_ in tires and shoe soles.

Besides CeO_2_-ENMs emitted from automotive exhaust systems, the majority of CeO_2_-ENMs resides within diesel particulate filters or the catalytic converters. In Germany, both elements of the exhaust system are shredded. A minor amount of the scrap is melted in a plasma furnace, where Ce remains bound in the slag because of its high oxygen affinity. A share of German scrap from automotive exhaust systems is exported and sold to international smelters (Source: own research and personal communication with scrap processing companies). Owing to the fact that the fraction of scrap from exhaust systems treated within Germany is unknown, as a simplifying assumption, the entire mass of residual CeO_2_-ENMs in exhaust systems is considered as a deposit to landfills.

According to Wang, *et al*.^57^, a large proportion of used tires is treated in waste incineration plants, including thermal utilization in cement production. Nevertheless, during use, one-tenth is released to soils. Whether SiO_2_-ENMs are released from tires and shoe soles mainly as single particles, agglomerates, or as a composite of rubber and ENM including other components like carbon black particles, is not known. Unless detailed information on the condition of emitted silica particles from rubber products becomes available, they have to be considered as single nanoparticles.

As mentioned above, owing to their varying toxicological effects, a differentiation between the types of silica is advisable. For use-based release into waste water, our modeling approach assigns the major contribution to precipitated silica. This dominant role of precipitated silica is attributable to the fact that it accounts for nearly three-quarters of the overall amount of produced silica (see Table S3) and has a number of applications with relevant releases into waste water (see Table S7). Pyrogenic silica, a form of silica with reported toxicological relevance, accounts only for a minor part of silica release into wastewater. Therefore, in an evaluation of associated risks (see below), we have to be aware that potential toxicologically relevant forms of silica may represent only a part of the total amount of engineered silica in particular compartments, at least for waterbodies and sediments. Moreover, it becomes clear that further work on exposure to ENMs has to consider the different applications of ENMs in more detail.

The broad range of our stochastic model outputs (Fig. 2) reflects uncertainty propagation due to mixing high uncertainty and variability of economic (ENM use) and scientific (release) data. Years of research to improve ENM use-release kinetics will be necessary to exceed the threshold of current preliminary and assumptive release data and subsequent risk modeling that only reflects homogeneous ENM dispersal in the environment at regional to continental levels. Normalizing mass flows to concentrations using total countrywide volumes has led to the impression that significant dilution occurs everywhere and without exception. This assumption may be applied in making preliminary and general predictions. However, it has significant limitations for understanding local, short-term, high-extreme exposure scenarios^31,54^. For example, considering CeO_2_ used for CMP implicates chemical and microelectronics industries near Dresden, which are on the Elbe River. Presumably, exposure in that region would be plausible in air, water, and agriculture. However, the residents of Hesse living along the Main might only experience air exposure caused by emissions from cars. In the United States, there is a mixing zone at the outfalls of all production plants. The U.S. Environmental Protection Agency (EPA) assumes a tenfold dilution and requests ecotoxicity data that are calculated at that concentration. These data must be available in order for the plants to receive effluent permits. We would have to quote that as the maximum concentration and then calculate the decrease in concentration caused by hetero-aggregation. This calculation would be similar to TiO_2_ on the Rhine^37^ or to Ag and ZnO nanoparticles modeling on the James River^40^.

The initial model validation, or, more realistically, the model-to-measurement comparisons shown above, is challenging. The possibility of statistical Type-III error cannot be fully excluded, owing to possibly having stochastically combined mostly non-validated input data to generate acceptable model output results. Furthermore, the available measurements do not distinguish between natural and engineered nanoparticles. Thus, the correlation between and the plausibility of the measurements and model results can be found by comparing the maximum upper limits of the modeling values, which should not exceed the measured equivalents. However, this correlation may also be found since wastewater should be dominated by engineered (non-natural) nanomaterial.

**Figure 7:**
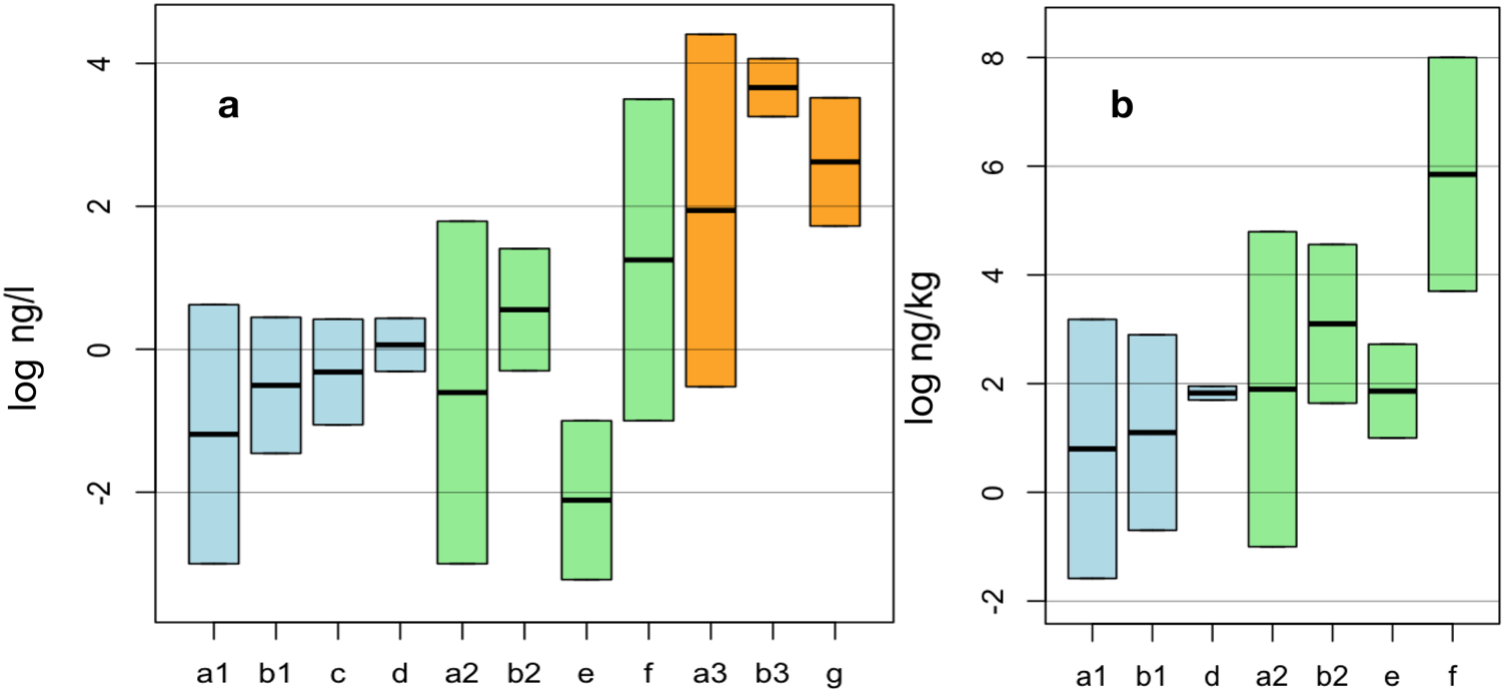
Comparisons of modeled PEC ranges (and means) for current values, logarithmically shown on the *y* axis for surface waters (Figure **a**) and soils (Figure **b**) (blue: Ag-ENMs; green: CeO_2_-ENMs; orange: SiO_2_-ENMs). Shown for all three materials are (**a1**–**a3**) the full range from our work, (**b1**–**b3**) our most probable range, (**c**) the full range of all PECs from the first probabilistic models^36^, (**d**) recent results^34^, (**e**) Denmark-specific PECs^77^, (**f**) results of a recent multimedia fate model^39^, and (**g**) silica-specific results^57^. Background colors are randomly chosen for clarity.

Comparing various modeling results has, in contrast to the comparison of measurements with modeling outputs, very low or no mutual validation power. Nevertheless, some limitations and plausibility may be discussed by opposing a non-comprehensive selection of other model results in Fig. 7 to our predictions for current concentrations in fresh water and soils. It is striking that we present the widest range of results, which speaks for our rigorous uncertainty treatment and integration of several years of dynamic release. As further evidence of this, previous results with similar model output ranges^34^, even if not showing the full range of Monte Carlo simulations, cover (albeit only in a rudimentary way) the full uncertainty found in the raw data currently available for time-dynamic uncertainty propagation. Our silver predictions fully agree with existing observations for waters and soils. Although we completely overhaul former modelling approaches that ignored time-dependent release variation and uncertainty, and although we use completely different data for different regions, our output ranges agree with values already published in the last decade^36^. This also reflects the short ENM residence time in water (lower than one year), which makes a dynamization for such receiving compartments almost superfluous. High agreement is also seen for CeO_2_ and SiO_2_ results, despite their being based on or reflective of various data, models, and investigated regions. A relatively large water volume and a lower ENM use leads to lower Danish CeO_2_-ENM water PECs^77^ that nevertheless fully lie in our range. Others^39^ have shown, for Europe, water PECs within our German range. However, the higher maximal values are not discussed in detail here because they partially exceed the relatively high concentrations measured in industrial wastewaters. Agreement is shown for soils, with the exception of high values reported in Meesters, *et al*.^39^. The silica-specific comparison with Wang, *et al*.^57^ shows good correlation; however, the reported range is narrower than our predicted range, owing to treating uncertainty in decades-spanning data less comprehensively as we do in our dynamic release modeling.

Modeling remains indispensable as long as analytical methods cannot distinguish background and engineered material. Preliminary environmental release, exposure, and risk assessment still leave room for at least partially misleading or erroneous findings. Thus, future preliminary risk estimation will be focusing on filling significant data gaps on ENM use and release amounts and overcoming significant modeling simplifications of non-mechanistic environmental fate analysis (dissolution, aggregation, sedimentation and other).

Closing the gap is a challenge. Industry has a reluctance to provide information for both competitive and anti-trust reasons, but also wishes to have ecotoxicity testing done at relevant exposure levels. The absence of this information has led some countries in Europe to institute chemical registries, which may broadcast production volumes of suppliers in those countries to their competitors. Some method of aggregating product statistics, perhaps monitored by trade associations and government agencies is yet to be found. Industrial data are needed on precise, current ENM mass production and use. Such data can be fed into more sophisticated economic nanoproduct consumption models. Even if such data become available, however, future ENM market dynamics will be difficult to predict, owing to their dependence on yet-unknown economic trends and regulative interventions^4^. Refined ENM use models should be linked to robust nanoproducts experimental evidence and should be based on the most relevant available environmental release paths, kinetics, and material forms. Release from nanoproducts will have to account for a wide material spectrum ranging from pristine particles to fragmented products^6^.

Finally, uniformly distributed mass flows in target systems on the country level only rudimentarily represent nanomaterial fate in the environment. In particular, spikes in daily and other short-term and locally specific exposure and associated potential risks are not covered by simplified, annually resolved computations with ENM distributions homogenized to the country level. Garner, *et al*.^31^ refined multi-media fate modeling by accounting for locally and time-resolved intercompartmental ENM transfers and transformations distinguishing free, homo- and heteroaggregated, as well as dissolved, nanomaterial. The authors point the way to holistically and mechanistically considering the most significant processes. Those include, among others, ENM attachment on aerosols, advection and sedimentation in air, erosion and runoff during rain events, dissolution and heteroaggregation, suspensions, resuspension and burial in waters and sediments, and water and sediment flow into lakes and sea water.

A remaining question is to what extent ENM ecotox and subsequent environmental risk modeling is impaired by the use of inconsistent toxicity data. The challenge regarding highly uncertain or contradictory data for nanotoxic endpoints has recently been reviewed^32^. The most critical issues were shown to be the absence of nano-specific effects that may either not have existed or that may have been masked by practical challenges to test performance on the nanoscale, such as “high-doses” material (nanomaterial) that was not fully characterized. Last but not least, an extreme variation of test methods was found, combined with unknown or unreported nanomaterial properties (particle, size distribution, aggregation dynamics, etc.) and missing reference or control samples for the performed tests. While such critical review made many valid points, it did not address the uncertainty when ‘official testing’ requirements, those protocols established by the OECD or national bodies, actually require modification and lead to situations where nominally thorough testing can still be incomplete.

## Methods

### Materials and Data

Based on a standardized questionnaire, a survey was conducted to assess the annually produced volume of SiO_2_-, CeO_2_-, and Ag-ENMs and their annual trends. 72 producers, dealers, and research institutions in the field of nanomaterials were contacted in the course of the survey. Despite repeated inquiry, useful information was hard to obtain. Of the 28 replying addressees, 20 refused to respond, either because of ignorance with respect to production volumes and trends or because of a restrictive company policy that prohibits giving any information or ratings in surveys. Eight questionnaires were completed. Six of these eight estimates turned out to be useful for further evaluation (cp. Figs S1a and S2). One of the six remaining addressees is a research institution; the other respondents are trading companies or producers of ENMs.

The share of the German annual market volume of the investigated ENMs was calculated by multiplying the global annual production of the respective ENMs by the ratio of German annual domestic demand and the worldwide gross domestic product (GDP) (in market prices). Using the German domestic demand instead of the German GDP corrects influences of annual export and import volumes.

For the collection of data on ENM-containing products and categorization of ENM applications, a number of publicly available databases were used, as listed in the Supplementary Information (chapter seven).

For the derivation of mass fractions according to product categories of the examined ENMs, see chapter two of the Supplementary Information. Estimations for the start of use of ENMs are based on the results of a literature search.

Product lifespan and TCs for the release of ENMs to natural and technical compartments were assigned as described in chapter three of the Supplementary Information. Assumptions are based on results of our own investigations, data given in recent publications, databases of the German Department of Statistics, and ECHA’s (European Chemicals Agency) environmental release categories^78^.

Transfer coefficients for the fate of ENMs in technical systems and natural compartments are listed in the Supplementary Information, in chapters four and five, respectively. Data on German geographic and aquatic conditions, as well as statistical information on the German waste management system, are given in chapter six of the Supplementary Information. All other data collected and generated are included in this article and its Supplementary Information.

### Release modeling for use and end-of-life material lifecycle phases

In order to provide comprehensive insight into the possible environmental exposure to the target ENMs, a full and time-dynamic lifecycle release spectrum from all currently known significant ENM use categories was modeled. As shown in Fig. 1, such modeling includes annual ENM mass input into the target system from domestic production or import of pure ENMs or products containing ENMs. This input is followed by mass flows into lifecycle phases of ENM product use, with or without ENM release to natural or technical environments. From such use phases, lifecycle time dependent ENM release to waters, wastewaters (and sewage treatment), soils, and air is modeled. Non-released ENMs at the end of such product use phases become EOL material treated in waste incineration, landfill, or recycling processes. Thus, the target system is never in equilibrium but constantly changes. A comprehensive inventory of the used ENM mass input and release coefficient parameters, including their variation and uncertainty ranges, are given in the Supplementary Information. Such dynamic ENM release was modeled for the time period 1950 to 2050. Still, as listed in the Supplementary Information, most ENM use started at the end of the last or in this century. The Supplementary Information also shows our determination, for all use categories, of the receiving compartments to which release of ENMs takes place. Such release is shown by equation 1 for a first scenario with short residence time of the emitted ENM.

1 $${r}_{1}(t)=\sum _{i}\sum _{j}{I}_{i}({t}_{j})$$

The release *r*_1_ reflects the modeled release at time *t* as a material input (I) function of all ENM-releasing engineered nanomaterial applications *i* and their life cycle years *j* contributing to such release. The life cycle length *m* (equations 3–6) reflects the whole release period. Such release leads to non-cumulative release and exposure of ENM in waters, air, sediments, and soils characterized by a short residence time (up to one year) before being degraded, eliminated, or transformed into other compounds. In contrast to this first scenario of short residence times of ENMs in nature, we also modeled (equation 2) an opposite scenario on the other side of the fate spectrum, with ENM persistently remaining in soils and sediments with no degradation.

2 $${r}_{2}(t)=\sum _{s}{r}_{1}({t}_{s})$$

This contrasting scenario reflects the same model input as given in the first scenario, but this time all computed material release (inputs [I]) from previous years are considered as well. That is of importance when the already released potential contaminant persistently remains in a particular natural or technical environment.

The *R-*based^79^ Monte Carlo implementation of such time-dynamic mass inputs and stochastic release from various applications into different ENM-receiving compartments is given in Fig. S1 and shown in detail elsewhere^58^. It allows stochastic exploration for all parameters of a wide range of possible values throughout the whole target ENM transfer system in time and space dimensions. Such transfer reflects unidirectional ENM transport, as done on other occasions, based on probabilistic material flow analysis designed for complex multidirectional transfer systems^36,80^.

### Use release

The use-release model covers release during the whole ENM lifecycle before ending up in EOL treatment such as waste disposal, recycling, or landfilling. Hence, such use-phase release occurs during the nanomaterial engineering and manufacturing of products (including import of material and products) and the consumption phase of those products. This model considers, for each application (product) as shown in equation 3, the annual release at a particular time (particular year during the whole lifecycle of a target ENM application) by considering all release contributions coming from all relevant lifecycle years up to that time point.

3 $${r}_{use}(t)=\sum _{j}\frac{I({t}_{j})}{m}$$

Equation 3 is derived from equation 1 (equivalent derivations for equation 2 are not shown) and didactically conceptualizes a regular (annual) time-dynamic release input for one single product use at time *t* for all its release-contributing product lifecycle years *j* up to time point *t* with *m* as lifecycle length of the corresponding product.

An additional use model reflecting the time-dynamic input into ENMs in circulation during use phases without any release (delayed release) has been modeled as well (equation 4). Such material in circulation mass covers the not-yet-released material at a certain time. This model considers all lifecycle years *j* not yet contributing to ENM release.

4 $${r}_{delayed(use)}(t)=\sum _{j}I({t}_{j})\cdot \frac{(m+j-t-1)}{m}$$

In addition to those use-release and use-in-circulation computations, we also modeled material no longer in consumption and release processes but already in nature (or in the technical sinks after EOL treatment (disposal, recycling, landfilling)). Such modeling may be derived from the release models by considering the past release periods or computed with their own input functions, as shown in conceptual equation S1 in the Supplementary Information.

### End of life release

EOL release covers, for each particular application, the ENMs not emitted during its whole product use life. That kind of release occurs, as shown in equation 5, at the end of the product life time *m*.

5 $${r}_{eol}(t)=I({t}_{t-m})\,\,\,({\rm{t}} > {\rm{m}})$$

For the EOL release modeling, the material in circulation (equation 6) refers to the ENMs in products not ending up in use-based release and waiting for EOL treatment via incineration, landfill, or recycling.

6 $${r}_{delayed(eol)}(t)=\sum _{j}I({t}_{j})\,({\rm{t}}\le {\rm{m}});$$

7 $${r}_{delayed(eol)}(t)=\sum _{j=t-m+1}I({t}_{j})\,({\rm{m}} < {\rm{t}}\le 2\,{\rm{m}})$$

Additionally, in the case of EOL modeling, ENMs already released (into nature or technical sinks) can be derived from the release computations by considering past release periods or from their own input functions (equation S2 in the Supplementary Information).

### Ecotoxicological and risk estimations

Risk has been estimated by comparing our environmental predictions to ecotoxicologically significant concentrations that can be derived from species sensitivity distribution (SSD) modeling. For water environments, some data were available for conducting PSSD analysis^35^, an approach allowing integration of highly controversial ecotoxicologial data for ENMs^27,57,81^. For the terrestrial compartments, PSSD could not be performed, because the data are too scarce and are even more uncertain than those for aquatic compartments.

To cope with the challenges of deriving and handling highly uncertain and controversial toxic endpoints, the Monte Carlo–based PSSD approach was used^35^. Such modeling does not eliminate uncertainties, but it stochastically explores a large spectrum of possible target toxic endpoints. In contrast to SSD methods that use a mean endpoint value for each single species, our PSSD develops a unique, single-species sensitivity distribution for each tested organism. If taken together, from all single-species distributions of all other tested organisms, the generic PSSD for a target ecosystem emerges. For the Ag- and CeO_2_-ENMs, we based our toxicological modeling on the endpoints taken from Holten Luetzhøft, *et al*.^82^, in the “Values” column of their Tables 17 and 21. For the SiO_2_-ENMs, the no-effect concentration endpoints are derived from Wang, *et al*.^57^, considering their “Concentration” column of the Table S7.

We applied a 50% uncertainty/variability interval around all endpoints fed into the ecotoxicology model. The same uncertainty/variability interval was applied around the factors (uncertainty factors 10) used to transform acute into chronic effects and the observed effects into no-effect predictions.

## Electronic supplementary material

Supplementary information
