# Supplementary material for: Risks, Release and Concentrations of Engineered Nanomaterial in the Environment
Source: Sci Rep. 2018 Jan 25;8:1565. doi: 10.1038/s41598-018-19275-4 (PMC5785520; doi:10.1038/s41598-018-19275-4)
Supplement: Supplementary file 1 — Supplementary information [file 41598_2018_19275_MOESM1_ESM.pdf]

# Risks, Release and Concentrations of Engineered Nanomaterial in the Environment

**Bernd Giese[1,2], Fred Klaessig[3,4], Barry Park[5], Ralf Kaegi[6], Michael Steinfeldt[1], Henning Wigger[1,7], Arnim von Gleich[1] and Fadri Gottschalk[8*]**

[1] University of Bremen, Faculty of Production Engineering, Department of Technology Design and Technology Development, Badgasteiner Str. 1 28359 Bremen, Germany
[2] University of Natural Resources and Life Sciences, Institute of Safety and Risk Sciences, Borkowskigasse 4, 1190 Vienna, Austria
[3] Pennsylvania Bio Nano Systems, Doylestown, Pennsylvania 18901, United States
[4] Center for Environmental Implications of Nanotechnology (UC CEIN), University of California Santa Barbara, Santa Barbara, California 93106-5131, United States
[5] GBP Consulting Ltd, Purton, UK
[6] Eawag, Swiss Federal Institute of Aquatic Science and Technology, Überlandstrasse 133, 8600 Dübendorf, Switzerland
[7] Empa, Swiss Federal Laboratories for Materials Science and Technology, CH-9014 St. Gallen, Switzerland
[8] ETSS AG, Engineering, technical and scientific services, CH-7558 Strada, Switzerland

*fadri.gottschalk@etss.ch

# Supplementary Information

## 1. Engineered nanomaterial use/production amounts

### Current use/production amounts

The large variance in the data in Table S1a and Table S1b does not stand for natural variability but for the large uncertainty in the assessment. Normal production amount probability distributions were modeled, which have emerged from the data and their variability including specified weighting and natural limits of non-zero and non-negative values. Specifications greater than 1 were e.g. interpreted as 1.25 (a quarter over the specified number). The values in S1 a, b refer to aggregated/agglomerated ENM. The highly dispersed fraction is covered as well, however, cannot be quantified based on the current available data.

**Table S1 a: 2015 Estimates for global production based on own survey**

| Source | $SiO_2$-ENM in t/a | $CeO_2$-ENM in t/a | Ag-ENM in t/a |
|---|---|---|---|
| Company A | > 1,000,000 | 10,000-100,000 | 1-10 |
| Company B | ca. 3,000,000 | | |
| Company C | >100,000 | 1,000-10,000 | 10,000-100,000 |
| Company D | | | 1,000-10,000 |
| Institute A | >100,000 | 1,000-10,000 | 100-1,000 |

**Table S1 b: Global estimates from literature**

| Source | $SiO_2$-ENM in t/a | $CeO_2$-ENM in t/a | Ag-ENM in t/a |
|---|---|---|---|
| Piccinno, Gottschalk et al. (2012) | 55-55,000 | 5.5-550 | 5.5-550 |
| Sun, Gottschalk et al. (2014) | | | 5.5-500 |
| SRI (2010) cited in EC (2012) | 1,500,000 | 10,000 | 22 |
| Burkhardt, Englert et al. (2011) | | | 140 |
| Keller, McFerran et al. (2013), Future Markets (2012) cited in Lazareva and Keller (2014) | 82,500-95,000 | 7,500-10,000 | 360-450 |
| Future Markets (2014) cited in Pulit-Prociak and Banach (2016) | 185,000-1,400,000 | 880-1,400 | 135-420 |

The share of the German annual market volume of the investigated ENM was calculated by multiplying the global annual production of the respective ENM by the ratio of German domestic demand (2014) and the worldwide gross domestic product (GDP) (in market prices for 2014).

### Trends in use/production amounts

We modeled the use data trend in time running in relation to the 2015 use volumes by orienting ourselves on our survey results and combining them with other very rough time based growth dynamics given elsewhere (Ricardo Energy & Environment 2016). It is impossible to make any precise predictions or retrospective indications on the use trend. Thus, we restricted ourselves on very rough trend estimations without providing any complex time dependent use computations based on differentiated time dynamics, which are mostly just reflecting highly controversial non-validated market development data. A median annual growth of 5% (with 50% uncertainty/variability range on each side) was used for the time period after 2000 that best combines our own survey results with the ones for established materials given in elsewhere (Ricardo Energy & Environment 2016). A median value of 1% (with 50% uncertainty/variability range on each side) annual growth was assumed for single engineered nanomaterial applications for the time before 2000 by only roughly orienting ourselves on some silver ENM application trends discussed for medical use (Sun, Bornhöft et al. 2016).

**Table S 2: Raw data on engineered nanomaterial (ENM) production trends for established applications. a) own survey, b) according to Ricardo Energy & Environment (2016)**

| a) | $SiO_2$-ENM | $CeO_2$-ENM | Ag-ENM |
|---|---|---|---|
| **Company/Institution** | **% Trend in +/-** | **% Trend in +/-** | **% Trend in +/-** |
| Company A | growing | | |
| Company B | +3-4 | | |
| Company C | +3 | +5 | +3 |
| Company D | | | +18 |

| b) period | % established materials |
|---|---|
| 2000-01 | 5 |
| 2001-05 | 10 |
| 2005-15 | 5 |
| 2015-36 | 10 |

## 2. Engineered nanomaterial applications

Table S3-5: Engineered nanomaterial environmental application fields described as product categories. The given values represent our best mass fraction estimations of a particular engineered nanomaterial used for a particular product category. These values were decreased and enlarged by 50% leading to triangular and symmetrical probability distributions. The distributional symmetry may be limited in the Monte Carlo (MC) based computations by highest or lowest possible fractions of 1 and/or 0.

Mass fractions of $SiO_2$-ENM were adopted from a study of the European Centre for Ecotoxicology and Toxicology of Chemicals (ECETOC) (JACC 2006) on synthetic amorphous silica and complemented with “lubricants, greases, release agents” according to Wang, Kalinina et al. (2016) and a predicted share based on own investigation.

For product categories of $CeO_2$-ENM, mass fractions are based on estimates for global consumption of $CeO_2$-ENM which are derived from information given in literature and databases. The global mass of $CeO_2$-ENM in catalytic converters is based on the worldwide automobile production of cars and commercial vehicles for 2013 (OICA 2015), 80 g $CeO_2$ per converter (Bleiwas 2013) and a share of 85 % for cars and commercial vehicles equipped with a catalytic converter (own assumption).

The estimate for worldwide consumption of $CeO_2$-ENM as diesel-fuel additive was calculated as a mean of high and low concentrations of $CeO_2$-ENM in diesel fuel (2-5 mg/l according to Johnson and Park (2012)) with a fuel consumption of 1000 l per year and a global number of 5 Million cars with a fuel born catalyst system (Rocher, Seguelong et al. 2011).

The global mass of $CeO_2$-ENM for use in exterior coatings was derived from (Gottschalk, Nowack et al. 2015). After scaling from the Danish to the German market (according to the ratio in population numbers), global consumption for exterior coatings was calculated by a factor which reflects the ratio of the worldwide gross domestic product (Worldbank 2016) to the German domestic demand (Statistisches Bundesamt 2016).

Global mass of $CeO_2$-ENM for use as polishing agent is based on numbers given by Goonan (2011) for glass polishing and Reed, Cormack et al. (2014) for chemical mechanical polishing (CMP). The worldwide amount of $CeO_2$ for use in NiMH-Batteries is based on information given in Goonan (2011).

Mass fractions of product categories containing Ag-ENM are based on the ratio of the overall market volumes of these categories and the mean share of Ag-ENM in corresponding products.

**Table S 3: $SiO_2$-ENM applications (product categories)**

| $SiO_2$-ENM | Fraction |
|---|---|
| **pyrogenic silica** | **0.148** |
| silicones - elastomers | 0.059 |
| polyester resin | 0.016 |
| epoxy resin | 0.010 |
| adhesives and sealants | 0.010 |
| paints and coatings | 0.022 |
| industrial inks | 0.006 |
| silicones – other | 0.010 |
| non-silicones - other (pharmaceutical, cosmetics, toner, batteries, food) | 0.013 |
| lubricants, greases, release agents | 0.001 |
| **colloidal silica** | **0.047** |
| refractory binders | 0.010 |
| investment casting | 0.008 |
| pulp and paper | 0.011 |
| textile processing | 0.005 |
| electronics | 0.004 |
| wine, beer, fruit juice, clarification | 0.005 |
| others | 0.004 |
| **gel silica** | **0.065** |
| plastics | 0.006 |
| paints and coatings | 0.010 |
| catalysts | 0.002 |
| textiles | 0.005 |
| gas drying | 0.002 |
| pharmaceutical tableting additive | 0.008 |
| food industry | 0.026 |
| dentifrices | 0.002 |
| absorbents and desiccants | 0.003 |
| **precipitated silica** | **0.745** |
| green tire | 0.119 |
| shoe soles | 0.112 |
| general tire | 0.075 |
| polymers/plastics | 0.060 |
| sealants and mastics | 0.037 |
| other rubber goods | 0.134 |
| paints, coatings, printing inks | 0.030 |
| toothpaste/cosmetics | 0.075 |
| carrier material | 0.067 |
| other | 0.037 |

The rounded values do not reflect accuracy; these are generated by mixing own assumptions and values from the literature given into a standardization of all values as a result of mass balances, which adds up for non-rounded figures to 100%.

Fractions derived are based on JACC (2006), Wang, Kalinina et al. (2016), and information from a producer of lubricants.

**Table S 4: $CeO_2$-ENM applications (product categories)**

| $CeO_2$-ENM | Fraction |
|---|---|
| catalytic converters | 0.283 |
| fuel additive | 0.001 |
| exterior coatings (among others wood) | 0.005 |
| polishes for glass and silicon wafers | 0.519 |
| NiMH batteries | 0.169 |
| automotive NiMH-batteries | 0,023 |
| further and unspecified categories with no relevant environmental release | no data available |

See please for comments on rounded values table S4.

**Table S 5: Ag-ENM applications (product categories)**

| Ag-ENM | Fraction |
|---|---|
| cleaning Agents | 0.0053 |
| coatings | 0.0062 |
| cosmetics | 0.0042 |
| medicine | 0.0121 |
| plastics | 0.0051 |
| textiles | 0.3099 |
| food & drink | 0.0002 |
| consumer electronics | 0.6471 |
| other applications (e.g., glass, filter) | 0.0100 |

See please for comments on rounded values table S4.

Fractions derived are based on information of market volumes in IKW (2015) for cleaning agents; VdL (2016) for coatings; Statistisches Bundesamt 2012 and Eurostat 2012 cited in Meo (2014) for cosmetics; STATISTA (2014) for plastics; Wigger, Hackmann et al. (2015) for textiles; BAH (2015) for Food & Drink. The mean share of Ag-ENM in product categories is based on information given in: Reidy, Haase et al. (2013), Wasukan, Srisung et al. (2015) and PEN Silver Database (2014) for cleaning agents; Kaegi, Sinnet et al. (2010) cited in Reidy, Haase et al. (2013) for coatings; Wasukan, Srisung et al. (2015) and PEN Silver Database (2014) for Cosmetics as well as medical applications; Mackevica, Olsson et al. (2016) for plastics; Burkhardt, Englert et al. (2011) for textiles and PEN Silver Database (2014) for food and drink.

## 3. Engineered nanomaterial environmental release

Table S6-S9: Engineered nanomaterial environmental release dynamics are presented in simplified form based on ENM product life time based Transfer Coefficients (TC) representing the fraction of engineered nanomaterial transported into natural or technical compartments. The given modal values were decreased and enlarged by 50% leading to triangular and symmetrical probability distributions (this does not apply to values of normal distributions). The distributional symmetry may be limited by highest or lowest possible fractions of 1 and/or 0. Use release (USE): USE release involves first early USE release that represents the ENM fraction released during the first year of the product use representing high initial releases due to loss of loosely incorporated ENM (within the product matrix). The subsequently released fractions are e.g. evenly distributed over the life cycle time remaining after this first year of. A more sophisticated, precise USE release differentiation is not useful because there is almost no validated empirical data that supports such precision. Insofar, we remain on very simple assumptions in our USE based release time dynamics. End of life release (EOL): The annual ENM transfers into product disposal processes are modeled at the end of each product life cycle.

Assumptions for the product lifespan are oriented on a classification given in Ricardo Energy & Environment (2016). ENM release during their production, formulation and manufacturing processes (Table S6) occurred before entering into our USE and EOL release phases.

**Table S 6: Engineered nanomaterial environmental release during production, formulation and manufacturing processes.**

| Categories | Release | Emission vector | | |
|---|---|---|---|---|
| | % emitted during production and manufacturing | % to air | % to waste water | % to waste disposal |
| suspensions for cosmetics, pharmaceuticals, pigments, paints, lacquers, inks, fuel additive adhesives, food additives, toner etc. | 1-5% | 10% | 30% | 60% |
| composites, plastics, construction material, polymers etc. | 0-1% | 0% | 5% | (50% recycling) 95% |
| electronic devices, batteries, catalytic converters etc. | 0-1% | 0% | 5% | (50% recycling) 95% |
| other categories: textiles, paper etc. | 0-1% | 1% | 1% | 99% |
| Done | | 0.001 | 0.003 | 0.005 |

The release factors in Table S6 are own assumptions that loosely follow release factors during production and manufacturing used in Gottschalk, Lassen et al. (2015) and for the allocation of this total release into different emission vectors we loosely used values given elsewhere (Ricardo Energy & Environment 2016).

**Table S 7: Engineered nanomaterial ($SiO_2$-ENM) environmental release. The ENM receiving fractions for Air, Surface water, Waste water and Soils equal to release during the ENM product use (USE release). The corresponding end of life (EOL) fractions go into Recycling, Waste incineration and Landfills.**

| | Product category | Start of use | Product lifespan (years) | | Early use release fraction | Air | Surface water | Waste water | Soils | Recycling | Waste incineration | Landfills |
|---|---|---|---|---|---|---|---|---|---|---|---|---|
| Pyrogenic silica | silicones – elastomers[1] | 1950 | 10 | 3σ=5 | e.o.t.t. | | 0.05 | 0.05 | 0.1 | 0.4 | | 0.4 |
| | polyester resin[2] | | 10 | 3σ=5 | e.o.t.t. | | 0.02500 | 0.2 | | | 0.75 | 0.025 |
| | epoxy resin[2] | | 10 | 3σ=5 | e.o.t.t. | | 0.025 | 0.2 | | | 0.75 | 0.025 |
| | adhesives and sealants[2] | | 10 | 3σ=5 | e.o.t.t. | | 0.025 | 0.2 | | | 0.75 | 0.025 |
| | paints and coatings[14] | | 10 | 3σ=5 | 0.9 | | 0.05 | 0.05 | 0.1 | 0.4 | | 0.4 |
| | industrial inks[24] | | 5 | 3σ=3 | 0.0 | | | | | 0.72 | 0.07 | 0.21 |
| | silicones – other[1] | | 30 | 3σ=10 | e.o.t.t. | | 0.05 | 0.05 | 0.1 | 0.4 | | 0.4 |
| | non-silicones – other[3] (pharmaceutical, cosmetics, toner, batteries, food) | | 1 | | 1.0 | | 0.1 | 0.75 | | 0.1 | 0.02 | 0.03 |
| | lubricants, greases, release agents[4,5] | | 5 | 3σ=3 | e.o.t.t. | 0.0005 | | 0.009 | 0.0005 | 0.85 | 0.2 | |
| Colloidal silica | refractory binders[6] | 1960 | 30 | 3σ=10 | | | | 0.01 | | 0.59 | 0.15 | 0.25 |
| | investment casting[7] | | 1 | | 1.0 | 0.01 | 0.01 | 0.01 | 0.01 | 0.9 | | 0.06 |
| | pulp and paper[8] | | 1 | | 1.0 | | | | | 0.8 | 0.1 | 0.1 |
| | textile processing[25] | | 1 | | 1.0 | 0.05 | | 0.05 | | | 0.459 | 0.441 |
| | electronics[10] | | 10 | 3σ=5 | e.o.t.t. | | | | | 0.7 | 0.05 | 0.25 |
| | wine, beer, fruit juice, clarification[11] | | 1 | | 1.0 | | | 1.0 | | | | |
| | other colloidal silica[23] | | 1 | | 1.0 | | 0.333 | 0.333 | 0.333 | | | |
| Gel silica | plastics[12] | 1960 | 10 | 3σ=5 | e.o.t.t. | 0.0025 | | 0.0125 | 0.235 | 0.604 | 0.075 | 0.072 |
| | paints and coatings[14] | | 10 | 3σ=5 | 0.9 | | 0.05 | 0.05 | 0.1 | 0.4 | | 0.4 |

| | | | | | | | | | | | | |
|---|---|---|---|---|---|---|---|---|---|---|---|---|
| | catalysts[15] | | 1 | | 1.0 | | | 0.05 | | 0.95 | | |
| | textiles[9] | | 5 | 3σ=3 | 0.5 | 0.25 | 0.03 | 0.58 | | 0.074 | 0.043 | 0.023 |
| | gas drying[16] | | 1 | | 1.0 | | 0.1 | 0.1 | | 0.4 | 0.204 | 0.196 |
| | pharmaceutical tableting additive[17] | | 1 | | 1.0 | | | 0.9 | | | 0.05 | 0.05 |
| | food industry[17] | | 1 | | 1.0 | | | 0.9 | | | 0.05 | 0.05 |
| | dentifrices[18] | | 1 | | 1.0 | | 0.1 | 0.85 | | | 0.02 | 0.03 |
| | absorbents and desiccants[19] | | 1 | | 1.0 | | 0.333 | 0.333 | 0.333 | | | |
| **Precipitated silica** | green tire[20] | 1990 | 5 | 3σ=3 | e.o.t.t. | 0.0001 | 0.001 | | 0.11 | 0.17 | 0.717 | |
| | shoe soles[20] | | 5 | 3σ=3 | e.o.t.t. | 0.0001 | 0.001 | | 0.11 | 0.17 | 0.717 | |
| | general tire[20] | | 5 | 3σ=3 | e.o.t.t. | 0.0001 | 0.001 | | 0.11 | 0.17 | 0.717 | |
| | polymers/plastics[12] | | 10 | 3σ=5 | e.o.t.t. | 0.0025 | | 0.0125 | 0.235 | 0.6 | 0.075 | 0.0717 |
| | sealants and mastics[2] | | 10 | 3σ=5 | e.o.t.t. | | 0.025 | 0.2 | | | 0.75 | 0.025 |
| | other rubber goods[1] | | 10 | 3σ=5 | e.o.t.t. | | 0.05 | 0.05 | 0.1 | 0.4 | | 0.4 |
| | paints, coatings, printing inks[21] | | 10 | 3σ=5 | 0.9 | | | 0.05 | | 0.9 | 0.02 | 0.03 |
| | toothpaste/cosmetics[18] | | 1 | | 1.0 | | 0.1 | 0.85 | | | 0.02 | 0.03 |
| | carrier material[22] | | 1 | | 1.0 | | 0.333 | 0.333 | 0.333 | | 0.0005 | 0.0005 |
| | other precipitated silica[22] | | 1 | | 1.0 | | 0.333 | 0.333 | 0.333 | | | |

e.o.t.t. = evenly distributed over total time
See please for comments on rounded values table S4.

[1] Release according to Wang, Kalinina et al. (2016) for "Putties, plasters, modeling clay".
[2] Release according to Wang, Kalinina et al. (2016) for "Adhesive and sealants".
[3] Grouping of “non-silicones – other” in accordance with CEFIC-ASASP, 2002 cited in European Commission 2007, p. 260. Own assumption for RF based on Wang, Kalinina et al. (2016) for "Cosmetics, personal care products".
[4] Release according to Keller, McFerran et al. (2013) for "Automotive".
[5] Release according to Wang, Kalinina et al. (2016) for “Lubricants, greases, release agents”.
[6] Release according to Wang, Kalinina et al. (2016) for “Stone, cement, glass and ceramics”.
[7] Own assumption based on Svehla, Krutzler et al. (2012), p.10.
[8] According to Sun, Gottschalk et al. (2014) for release of Nano-TiO2 from “Paper”.

[9] Own assumption based on Wigger, Hackmann et al. (2015).
[10] Own assumption according to Wang, Kalinina et al. (2016) for "Electrical / electronic products".
[11] Release according to JACC (2006) for "Wine, beer, fruit juice, clarification".
[12] Release to air, surface water, sewage treatment plant and soil according to Keller, McFerran et al. (2013) for "plastics". Release of plastic from household waste to WIP and landfill as well as share of recycling according to German waste statistics for 2014 (Statistisches Bundesamt 2016).
[14] Release according to Wang, Kalinina et al. (2016) for "Paints".
[15] Release according to Wang, Kalinina et al. (2016) for "Catalysts".
[16] Own assumption based on JACC (2006) for "Gas drying".
[17] Release according to Wang, Kalinina et al. (2016) for "Food products".
[18] Release according to Wang, Kalinina et al. (2016) for "Cosmetics, personal care products".
[19] Own assumption based on JACC (2006) for "Absorbents and desiccants".
[20] Own assumption based on Wang, Kalinina et al. (2016) for "Car tires" (WIP includes cement production).
[21] Release according to Wang, Kalinina et al. (2016) for "Coatings".
[22] Own assumption based on JACC (2006) for "Carrier material".
[23] Own assumption based on JACC (2006) for "colloidal silica – Others".
[24] Release according to Wang, Kalinina et al. (2016) for "Inks and toners".
[25] Own assumption based on ECHA's Environmental Release Category (ERC, Version 3.0) No. 12c "Use of articles at industrial sites with low release" and overall relation of German incinerated and landfilled waste (Statistisches Bundesamt 2016).

**Table S 8: Engineered nanomaterial ($CeO_2$-ENM) environmental release. The ENM receiving fractions for Air, Surface water, Waste water and Soils equal to release during the ENM product use (USE release). The corresponding end of life (EOL) fractions go into Recycling, Waste incineration and Landfills.**

| | Product category | Start of use | Life time (years) | | Early use release fraction | Air | Surface water | Waste water | Soils | Recycling | Waste incineration | Landfills |
|---|---|---|---|---|---|---|---|---|---|---|---|---|
| $CeO_2$-ENM | automotive catalytic converters[1] | 1980 | 10 | 3σ=5 | e.o.t.t. | 0.0053 | | | | | | 0.99 |
| | diesel fuel additive[2] | 2000 | 1 | | 1.0 | 0.05 | | | | | | 0.95 |
| | exterior paints (e.g., for wood)[3] | 2000 | 10 | 3σ=5 | 0.9 | 0.00008 | 0.0004 | 0.0048 | 0.0028 | | 0.992 | |
| | chemical-mechanical polishing (CMP) of glass and silicon wafers[4] | 1990 | 1 | | 1.0 | 0.025 | | 0.025 | 0.025 | | 0.472 | 0.453 |
| | NiMH-batteries[5] | 1990 | 1 | | 1.0 | 0.0005 | 0.016 | 0.016 | 0.032 | | 0.477 | 0.458 |
| | automotive NiMH-batteries[6] | 2000 | 5 | 3σ=3 | e.o.t.t. | | | | | 1 | | |

e.o.t.t. = evenly distributed over total time
See please for comments on rounded values table S4.

[1] Own assumption based on release of platin group metals (PGM) from automotive catalytic converters according to Helmers (1997). According to Öko-Institut there is no recycling of rare earth metals (REM) from automotive catalytic converters. REM remain in the slag during remelt-processes because of their high oxygen affinity. (Schüler, Buchert et al. 2011, p. 108)
[2] Own assumption based on Johnson and Park (2012). According to automotive recyclers (personal communication) there is up to now no recycling of $CeO_2$ from soot in diesel particulate filters (DPF). REM remain in the slag during remelt-processes because of their high oxygen affinity (Schüler, Buchert et al. 2011, p. 108).
[3] Own assumption based on Keller, McFerran et al. (2013) for “Coatings, Paints, & Pigments” and Scifo, Chaurand et al. (2014). Relevant fractions of household waste are recycled thermally (Statistisches Bundesamt 2016).
[4] Own assumption based on ECHA’s Environmental Release Category (ERC, Version 3.0) No. 12a “Processing of articles at industrial sites with low release”, information from Öko-Institut (Schüler, Buchert et al. 2011, p.108) for recycling and overall relation of German incinerated and landfilled waste (Statistisches Bundesamt 2016).
[5] Own assumption based on ECHA’s Environmental Release Category (ERC, Version 3.0) No. 10a " Widespread use of articles with low release (outdoor)", information from Öko-Institut (Schüler, Buchert et al. 2011, p.108) for recycling and overall ratio of German incinerated to landfilled waste (Statistisches Bundesamt 2016).
[6] Own assumption.

**Table S 9: Engineered nanomaterial (Ag-ENM) environmental release. The ENM receiving fractions for Air, Surface water, Waste water and Soils equal to release during the ENM product use (USE release). The corresponding end of life (EOL) fractions go into Recycling, Waste incineration and Landfills.**

| | Product category | Start of use | Life time (years) | | Early use release fraction | Air | Surface water | Waste water | Soils | Recycling | Waste incineration | Landfills |
|---|---|---|---|---|---|---|---|---|---|---|---|---|
| Ag-ENM | cleaning agents[1] | 1990 | 1 | | 1.0 | 0.1 | 0.025 | 0.8 | 0.025 | | 0.05 | |
| | coatings[2] | 1990 | 10 | 3σ=5 | 0.9 | | | 0.05 | | 0.9 | 0.02 | 0.03 |
| | cosmetics[3] | 1990 | 1 | | 1.0 | | 0.1 | 0.85 | | | 0.02 | 0.03 |
| | medicine[4] | 1900 | 1 | | 1.0 | 0.0125 | | 0.225 | 0.0125 | | 0.383 | 0.37 |
| | plastics[5] | 1990 | 10 | 3σ=5 | e.o.t.t. | 0.0025 | | 0.0125 | 0.235 | 0.604 | 0.075 | 0.072 |
| | textiles[6] | 2000 | 5 | 3σ=4 | 0.5 | 0.25 | 0.03 | 0.58 | | 0.074 | 0.043 | 0.023 |
| | food & Drink[7] | 1990 | 1 | | 1.0 | | | 0.9 | | | 0.05 | 0.05 |
| | electronics[8] | 1990 | 10 | 3σ=5 | e.o.t.t. | | | | | 0.7 | 0.05 | 0.25 |
| | other (e.g., glass, filter)[9] | 1990 | 10 | 3σ=5 | e.o.t.t | | | 0.01 | | 0.59 | 0.15 | 0.25 |

e.o.t.t. = evenly distributed over total time
See please for comments on rounded values table S4.

[1] Own assumption based on ECHA's Environmental Release Category (ERC, Version 3.0) No. 8d "Widespread use of non-reactive processing aid (no inclusion into or onto article, outdoor)" (ECHA 2016), Wang, Kalinina et al. (2016) for "Cleaning agents". According to German waste statistics relevant fractions of household waste are incinerated (Statistisches Bundesamt 2016).
[2] Release according to Wang, Kalinina et al. (2016) for "Coatings".
[3] Release according to Wang, Kalinina et al. (2016) for "Cosmetics, personal care products".
[4] Own assumption based on Keller, McFerran et al. (2013) for "Medical" and overall ratio of German incinerated to landfilled waste (Statistisches Bundesamt 2016).
[5] Release to air, surface water, sewage treatment plant and soil according to Keller, McFerran et al. (2013) for "plastics". Release of plastic from household waste to WIP and landfill as well as share of recycling according to German waste statistics (Statistisches Bundesamt 2016).
[6] Own assumption based on Wigger, Hackmann et al. (2015).
[7] Release according to Wang, Kalinina et al. (2016) for "Food products".
[8] Own assumption according to Wang, Kalinina et al. (2016) for "Electrical / electronic products".
[9] Own assumption according to Wang, Kalinina et al. (2016) for "Stone, cement, glass and ceramics".

## 4. Engineered nanomaterial fate in technical systems

The tables S 10-12 contain data on the engineered nanomaterial fate in technical systems represented in simplified form by Transfer Coefficients (TC). Those TCs stand for the annual fraction of ENM transport, transformation/elimination and/or deposition in or between technical systems. Single raw values were varied by ±50% for computing triangular and symmetrical probability distributions. Such symmetry may be affected by considering the limits of highest or lowest possible fraction values of 1 and 0. A value of 1 means that all ENMs are transported, transformed/eliminated and/or deposited and a value of 0 means that no ENM is involved in such processes.
In cases with several raw data values or for exceptions on single value sources, specific information on the probabilistic modeling procedure is given in each case.
Since data on ENM product export is not available, we did not integrate such export by performing a conservative release and environmental exposure modeling.

**Table S 10: Engineered nanomaterial ($SiO_2$-ENM) fate in technical compartments**

| **Sewage treatment plants (STP) and STP influents and effluents** | | |
|---|---|---|
| **Parameter** | **Transfer coefficient** | **Comments and data source** |
| STP connection rate (waste water to sewage treatment) | 0.96 (10 % variation on each side) | Sewage treatment plant connection rates indicated as fraction of total population (2013) (Statistisches Bundesamt 2016) |
| Waste water to surface water | 1- STP connection rate | |
| Sewage treatment plant overflow | 0.06 (0.05-0.3) | 6% (mode value) dirty water overflow in the catchment area of the Nahe (Schmitt, Knerr et al. 2016), and variation range according to oral information from the German Environment Agency (UBA) for dirty water overflow due to heavy rain. |
| Sewage water treated | 1- sewage treatment plant overflow | |
| Sewage treatment to sewage sludge (STP removal efficiency) | 0.94<br>0.97<br>0.818-0.927<br>0.99<br>0.9<br>0.988<br>0.96-0.993<br>0.945-0.961 | As listed in Wang, Kalinina et al. (2016):<br>Coated silica nanoparticles (Jarvie, Al-Obaidi et al. 2009)<br>DNA encapsulated silica nanoparticles (Grass, Schälchli et al. 2014)<br>Recovery of pure water flux (Pan, Huang et al. 2005)<br>Turbidity removal (Liu, Tourbin et al. 2013)<br>Turbidity removal (Den and Huang 2006)<br>Turbidity removal efficiency for electrocoagulation (Den and Huang 2005)<br>Turbidity removal efficiency for chemical coagulation (Den and Huang 2005)<br>Silica removal (Huang, Jiang et al. 2004)<br>Empirical probability distributions computed based on evenly bootstrapping of all listed values and ranges. |
| Transformation in sewage sludge | | Not considered. |
| Remaining in sewage sludge | 1- Transformation in sewage sludge | |

| | | |
|---|---|---|
| Sewage sludge disposed of to soils | 0.40 (10 % variation on each side) until incl. 2029<br><br>0.30 (10 % variation on each side) from 2030 - 2050 | Share of sewage sludge deposited on agricultural fields and for landscape construction measures (maximum amount 5 t applied within 3 years on 1 ha), average of the years 2012, 13, 14; own calculation based on (Genesis 2016)<br><br>According to the new regulation for sewage sludge in Germany, the amount of sludge deposited on agricultural soils or applied for landscape measures will be gradually decreased (Schneichel 2016, BMUB 2017). |
| Sewage sludge incinerated | 0.58 (10 % variation on each side) until incl. 2029<br><br>0.68 (10 % variation on each side) from 2030 - 2050 | Fraction incinerated (“thermische Entsorgung”), average of the years 2012, 13, 14; own calculation based on (Genesis 2016). We rounded up by neglecting 2% not further specified material utilization.<br><br>According to the new regulation for sewage sludge in Germany, the amount of sludge deposited on agricultural soils or applied for landscape measures will be gradually decreased (Schneichel 2016, BMUB 2017).<br>We rounded up by neglecting 2% not further specified material utilization. |
| Sewage sludge to landfills | 1991: 0.418<br>1995: 0.179<br>1998: 0.083<br>2001: 0.066<br>2004: 0.035<br>2006: 0.002<br>2007: 0.002<br>2008: 0.001<br>2009-2013: ~0 | Statistisches Jahrbuch des Bundesministeriums für Ernährung und Landwirtschaft (BMEL), (BMEL 2015, p. 88)<br><br>We assumed stable values before 1991. |
| Sewage treatment to sewage effluent | 1- Sewage treatment to sewage sludge | |
| Transformation in sewage effluent | | Not considered. |
| Sewage effluent to surface water | 1- Transformation in sewage effluent | |
| **Waste incineration plant (WIP) and WIP influents and effluents** | | |
| **Parameter** | **Transfer coefficient** | **Comments and data source** |
| Fraction of solid waste treated in incineration plants (WIP) | 0.17 (industrial and household) | Own calculations based on Destatis Umwelt - Abfallentsorgung, Fachserie 19, Reihe 1, Angaben für 2014 (Statistisches Bundesamt 2016) |
| Transformation during WIP and related processes (Incinerator, Boiler, Filter, Slag pool, Wet scrubber) into other forms | <0.0001 (low value) | See comments on data and data sources (Walser and Gottschalk 2014) for WIP related processes in Table S 11. |

| | | |
|---|---|---|
| WIP to slag | 0.75 (mean value) | Dito. |
| WIP to fly ash | 0.25 (mean value) | Dito. |
| WIP to waste water | 0.0001 (mean value) | Dito. |
| WIP to air | <0.0001 (mean value) | Dito. |
| Slag to recycling (construction works) | 0.31(road construction)<br><br>0,06 (use belowground)<br><br>0.09 (metals separation and other use) | (Alwast and Riemann 2010) |
| Slag to landfills | 0,54 | Dito. |
| Fly ash to recycling (cement) | 0.39 | As done elsewhere (Sun, Gottschalk et al. 2014) based on (Walser, Hellweg et al. 2012) |
| Fly ash to export | 0.22 | Dito. |
| Fly ash to landfills | 0.39 | Dito. |
| **Landfills (LAN) and LAN influents and effluents** | | |
| **Parameter** | **Transfer coefficient** | **Comments and data source** |
| Fraction of solid waste ending up in landfills (LAN) | 0.27 | Own calculations based on Destatis Umwelt - Abfallentsorgung, Fachserie 19, Reihe 1, Angaben für 2014 (Statistisches Bundesamt 2016) |
| Release from landfills to groundwater and surrounding surface waters | 0 | Data available for leaching out of landfills is currently not available. Zero emissions were assumed. In accordance to earlier work (Gottschalk, Lassen et al. 2015) we stopped the model at this nanomaterial life cycle stage by considering landfilling as ENM sink. |
| **Recycling (REC) and REC influents and effluents** | | |
| **Parameter** | **Transfer coefficient** | **Comments and data source** |
| Fraction of solid waste ending up in recycling processes (REC) | 0.56 (industrial and household) | Own calculations based on Destatis Umwelt - Abfallentsorgung, Fachserie 19, Reihe 1, Angaben für 2014 (Statistisches Bundesamt 2016) |
| REC to WIP | 0.2-0.85<br><br>(depending on the product category) | First data available in (Caballero-Guzman, Sun et al. 2015) depending on the product category containing the ENM. We very roughly orient ourselves on the Ag, ZnO and TiO2 ENM estimations:<br><br>consumer electronics 0.65-0.85, medtech 0.5, paints 0.2, for other categories 0. |

| | | |
|---|---|---|
| REC to LAN | 0-0.7 (depending on the product category) | Dito, for paints 0.68, for other categories 0. |
| REC to production, manufacturing, consumption (PMC) | 0-0.1 (depending on the product category) | Dito, for textiles 0.05, paints 0.08, for other categories 0.<br><br>Wigger, Hackmann et al. (2015) report appr. 0.011 for textiles. |
| REC to waste water | 0-0.2 (depending on the product category) | Dito, for consumer electronics 0.04-0.16, for other categories 0. |
| REC to transformation | 0-1 (depending on the product category) | Dito, for consumer electronics 0.07, medtech 0.5, metals 1, for other categories 0. |

**Table S 11: Engineered nanomaterial ($CeO_2$-ENM) fate in technical systems**

| **Sewage treatment plants (STP) and STP influents and effluents** | | |
|---|---|---|
| Parameter | Transfer coefficient | Comments and data source |
| STP connection rate (waste water to sewage treatment) | 0.96 (10 % variation on each side) | See comments on data and data sources for this STP parameter in Table S 10. |
| Waste water to surface water | 1- STP connection rate | |
| Sewage treatment plant overflow | 0.06 (0.05-0.3) | Dito. |
| Sewage water treated | 1- sewage treatment plant overflow | |
| Sewage treatment to sewage sludge (STP removal efficiency) | 0.95-98<br>0.97 | (Limbach, Bereiter et al. 2008, Gomez-Rivera, Field et al. 2012) |
| Transformation in sewage sludge | 33-48 | Ce(III) phase generated transformed ($Ce_2S_3$) (Barton, Auffan et al. 2014) |
| Remaining in sewage sludge | 1- Transformation in sewage sludge | |
| Sewage sludge disposed of to soils | 0.40 (10 % variation on each side) until incl. 2029<br><br>0.30 (10 % variation on each side) from 2030 - 2050 | See comments on data and data sources for this parameter in Table S 10. |

| | | |
|---|---|---|
| Sewage sludge incinerated | 0.58 (10 % variation on each side) until incl. 2029<br><br>0.68 (10 % variation on each side) from 2030 - 2050 | Dito. |
| Sewage sludge to landfills | 1991: 0.418<br>1995: 0.179<br>1998: 0.083<br>2001: 0.066<br>2004: 0.035<br>2006: 0.002<br>2007: 0.002<br>2008: 0.001<br>2009-2013: ~0 | Dito.<br><br>We assumed stable values before 1991. |
| Sewage treatment to sewage effluent | 1- Sewage treatment to sewage sludge | |
| Transformation in sewage effluent | 0-10 | (Barton, Auffan et al. 2014) |
| Sewage effluent to surface water | 1- Transformation in sewage effluent | |
| **Waste incineration plant (WIP) and WIP influents and effluents** | | |
| Parameter | Transfer coefficient | Comments and data source |
| Fraction of solid waste ending up in waste incineration plants (WIP) | 0.17 (industrial and household) | See comments on data and data sources for these parameters in Table S 10. |
| Transformation during WIP and related processes (Incinerator, Boiler, Filter, Slag pool, Wet scrubber) into other forms | <0.0001 (low value) | $CeO_2$ ENM fate in WIP is modelled following (Walser and Gottschalk 2014) that confirm earlier experimental findings (Walser, Limbach et al. 2012). Our parameters refer to figure 3 and 4a (Walser and Gottschalk 2014).This reference study provides a comprehensive uncertainty analysis of $CeO_2$ fate in WIP. Due to measurement limitations a significant part of the ENM cannot be detected analytically. If this not detected ENM $CeO_2$ fraction is transformed or deposited inside the plant, e.g. attached to the plant walls is currently unknown. We base our model on the very low metal and completely insignificant transformation fraction in WIP processes. Discriminating nano-$CeO_2$ transformed from deposited nano-$CeO_2$ inside the plant is currently not possible due to measurement limitations. Hence, not detected nano-$CeO_2$ has evenly been attributed in each treatment stage to subsequent transfer and/or deposition/transformation processes inside the WIP process. |
| WIP to slag | 0.75 (mean value) | Dito. |
| WIP to fly ash | 0.25 (mean value) | Dito. |

| | | |
|---|---|---|
| WIP to waste water | 0.0001 (mean value) | Dito. |
| WIP to air | <0.0001 (mean value) | Dito. |
| Slag to recycling (construction works) | 0.31(road construction)<br><br>0,06 (use belowground)<br><br>0.09 (metals separation and other use) | See comments on data and data sources for these parameters in Table S 10. |
| Slag to landfills | 0.54 | Dito. |
| Fly ash to recycling (cement) | ca. 0.50 | Dito. |
| Fly ash to landfills | ca. 0.50 | Dito. |
| **Landfills (LAN) and LAN influents and effluents** | | |
| Parameter | Transfer coefficient | Comments and data source |
| Fraction of solid waste ending up in landfills (LAN) | 0.27 | See comments on data and data sources for these LAN parameters in Table S 10. |
| Release from landfills to groundwater and surrounding surface waters | 0 | Dito. |
| **Recycling (REC) and REC influents and effluents** | | |
| Parameter | Transfer coefficient | Comments and data source |
| Fraction of solid waste ending up in recycling processes (REC) | 0.56 | See comments on data and data sources for these REC parameters in Table S 10. |
| REC to WIP | 0.2-0.85<br><br>(depending on the product category) | Dito, for consumer electronics 0.65-0.85, medtech 0.5, paints 0.2, for other categories 0. |
| REC to LAN | 0-0.7<br>(depending on the product category) | Dito, for paints 0.68, for other categories 0. |
| REC to production, manufacturing, consumption (PMC) | 0-0.1<br>(depending on the product category) | Dito, for textiles 0.05, paints 0.08, for other categories 0.<br><br>Wigger, Hackmann et al. (2015) suggest appr. 0.011 for textiles. |

| | | |
|---|---|---|
| REC to waste water | 0-0.2 (depending on the product category) | Dito, for consumer electronics 0.04-0.16, for other categories 0. |
| REC to transformation | 0-1 (depending on the product category) | Dito, for consumer electronics 0.07, medtech 0.5, metals 1, for other categories 0. |

**Table S 12: Engineered nanomaterial (Ag-ENM) fate in technical systems**

| **Sewage treatment plants (STP) and STP influents and effluents** | | |
|---|---|---|
| Parameter | Transfer coefficient | Comments and data source |
| STP connection rate (waste water to sewage treatment) | 0.96 (10 % variation on each side) | See comments on data and data sources for this STP parameter in Table S 11.<br><br>No losses of ENMs are expected in the wastewater collection system (Kaegi, Voegelin et al. 2011). |
| Waste water to surface water | 1- STP connection rate | |
| Sewage treatment plant overflow | 0.06 (0.05-0.3) | Dito. |
| Sewage water treated | 1- sewage treatment plant overflow | |
| Sewage treatment to sewage sludge (STP removal efficiency) | 0.95<br>0.975<br>0.8-1<br>1<br><br>0.99 | (Li, Hartmann et al. 2013)<br>(Kaegi, Voegelin et al. 2011)<br>for nano-Ag<br>for Ag+($AgNO_3$)<br>(Hedberg J., Baresel C. et al. 2014)<br>(Ma, Levard et al. 2014)<br>(Kaegi, Voegelina et al. 2013) |
| Transformation in sewage sludge | 0.98-1<br>0.94-1 | (Kaegi, Voegelin et al. 2011)<br>(Lombi, Donner et al. 2013, Ma, Levard et al. 2014) as referred in Sun, Bornhöft et al. (2016) |
| Remaining in sewage sludge | 1- Transformation in sewage sludge | |
| Sewage sludge disposed of to soils | 0.40 (10 % variation on each side) until incl. 2029<br><br>0.30 (10 % variation on each side) from 2030 - 2050 | See comments on data and data sources for this parameter in Table S 11. |
| Sewage sludge incinerated | 0.58 (10 % variation on each side) until incl. 2029<br><br>0.68 (10 % variation on each side) from 2030 - 2050 | Dito. |

| | | |
|---|---|---|
| Sewage sludge to landfills | 1991: 0.418<br>1995: 0.179<br>1998: 0.083<br>2001: 0.066<br>2004: 0.035<br>2006: 0.002<br>2007: 0.002<br>2008: 0.001<br>2009-2013: ~0 | Dito.<br><br>We assumed stable values before 1991. |
| Sewage treatment to sewage effluent | 1- Sewage treatment to sewage sludge | |
| Transformation in sewage effluent | 0.86-1 | (Kaegi, Voegelin et al. 2011) |
| Sewage effluent to surface water | 1- Transformation in sewage effluent | |
| **Waste incineration plant (WIP) and WIP influents and effluents** | | |
| Parameter | Transfer coefficient | Comments and data source |
| Fraction of solid waste ending up in waste incineration plants (WIP) | 0.17 (industrial and household) | See comments on data and data sources for these parameters in Table S 11. |
| Transformation during WIP and related processes (Incinerator, Boiler, Filter, Slag pool, Wet scrubber) into other forms | <0.0001 (low value) | See comments on data and data sources for these parameters in Table S 11. |
| WIP to slag | 0.75 (mean value) | Dito. |
| WIP to fly ash | 0.25 (mean value) | Dito. |
| WIP to waste water | 0.0001 (mean value) | Dito. |
| WIP to air | <0.0001 (mean value) | Dito. |
| Slag to recycling (construction works) | 0.31(road construction)<br><br>0,06 (use belowground)<br><br>0.09 (metals separation and other use) | See comments on data and data sources for these parameters in Table S 10. |
| Slag to landfills | 0.54 | Dito. |
| Fly ash to recycling (cement) | 0.39 | Dito. |

| | | |
|---|---|---|
| Fly ash to export | 0.22 | Dito. |
| Fly ash to landfills | 0.39 | Dito. |
| **Landfills (LAN) and LAN influents and effluents** | | |
| Parameter | Transfer coefficient | Comments and data source |
| Fraction of solid waste ending up in landfills (LAN) | 0.27 | See comments on data and data sources for these LAN parameters in Table S 10. |
| Release from landfills to groundwater and surrounding surface waters | 0 | Dito. |
| **Recycling (REC) and REC influents and effluents** | | |
| Parameter | Transfer coefficient | Comments and data source |
| Fraction of solid waste ending up in recycling processes (REC) | 0.56 (industrial and household) | See comments on data and data sources for these REC parameters in Table S 10. |
| REC to WIP | 0.2-0.85 (depending on the product category) | Dito, for consumer electronics 0.65-0.85, medtech 0.5, paints 0.2, for other categories 0. |
| REC to LAN | 0-0.7 (depending on the product category) | Dito, for paints 0.68, for other categories 0. |
| REC to production, manufacturing, consumption (PMC) | 0-0.1 (depending on the product category) | Dito, for textiles 0.05, paints 0.08, for other categories 0.<br><br>Wigger, Hackmann et al. (2015) report 0.011 for textiles. |
| REC to waste water | 0-0.2 (depending on the product category) | Dito, for consumer electronics 0.04-0.16, for other categories 0. |
| REC to transformation | 0-1 (depending on the product category) | Dito, for consumer electronics 0.07, medtech 0.5, metals 1, for other categories 0. |

## 5. Engineered nanomaterial fate in natural compartments

The table S 13 provides information on raw data or data gaps for the engineered nanomaterial fate in natural environments. The Transfer Coefficients (TC) represent as said before the annual fraction of engineered nanomaterial transport, transformation/elimination and/or deposition. Our best estimations were decreased and enlarged by 50% by means of triangular and symmetrical probability distributions limited by the highest or lowest possible fraction values of 1 and 0.

**Table S 13: Engineered nanomaterial fate in natural compartments.**

| **Surface water** | | |
|---|---|---|
| Parameter and compartment | Transfer coefficient | Comments and data source |
| Sedimentation from air | 1<br><br>(10 d/y atmospheric retention time) | We refer to earlier studies (Gottschalk, Sonderer et al. 2009, Sun, Conroy et al. 2015) that base their estimation on life-time data of ultrafine particles (Anastasio and Martin 2001) |
| Sedimentation from surface water | 0-1 | The full sedimentation processes spectrum has been modelled based on a highly complex inconclusive data situation (Praetorius, Scheringer et al. 2012, Praetorius, Gottschalk et al. under review). Thus, the sedimentation from the water was considered by exploring all events ranging from complete and immediate sedimentation to theoretically no sedimentation at all. |
| Soil-water transfer (erosion) | 0.006 | The estimation is based on data for erosion e.g. during storm events as used in earlier studies (Sun, Conroy et al. 2015) where such a value was derived from some evidence of such transport for linear alkylbenzene sulphonate (LAS) (Kannan, White et al. 2007, Sun, Conroy et al. 2015) |
| Dissolution in the water phase | $SiO_2$ ENM<br><br>$CeO_2$ ENM<br><br>Ag ENM | Not considered, due to currently missing evidence/lack of data (Wang, Kalinina et al. 2016).<br><br>Not accounted for because of lack of knowledge (Gottschalk, Lassen et al. 2015)<br><br>Accounted for in STP effluents but not quantified for the retention time in natural waters (Sun, Bornhöft et al. 2016). |
| Dissolution and other reactions in soils and in sediments. | | We stopped our model after the ENM enters the sediment and soil compartments. Currently there are no usable data available to subsequently perform such a highly complex environmental fate analysis. Thus, soils and sediments represent final sinks in the natural environment comparable to landfills in the technosphere. Insofar, we have to emphasize that our modelled concentrations in soils and sediments do not account for any nanomaterial dissolution or transformation that will likely be very significant for all three materials (Ag-ENM, $CeO_2$-ENM (Gottschalk, Lassen et al. 2015), and $SiO_2$-ENM (Wang, Kalinina et al. 2016) ). |

## 6. Geographic, aquatic and waste management data

Predicted environmental concentrations (PEC) have been derived by dividing the computed engineered nanomaterial flows and deposition volumes by the mass/volume of a particular environmental compartment (bulk material or technical compartment) receiving nanomaterial.

**Table S 14: Geographic, aquatic and waste handling data**

| Parameter | Unit | Value | Comments and data source |
|---|---|---|---|
| **Geographic data** | | | |
| Population of Germany | Inhabi-tants | 81,197,500 | German population 2014 according to (Statistisches Bundesamt 2016) |
| Total area of Germany | $km^2$ | 357,376 | German area 2014 (Statistisches Bundesamt 2015) |
| Height of the air compartment | km | 1 | Relevant atmospheric height (ECB 2003) |
| Area of natural soil | % | 32.19 | moorland (841 km2) + heathland (602 km2) + forest area (109,306 km2) + infertile land (4,281 km2), data of 2014 |
| Area of agricultural soil | % | 51.25 | farmland (incl. moorland and heathland) (184,607 km2) - moorland (841 km2) - heathland (602 km2), data of 2014 (Statistisches Bundesamt 2015) |
| Area of urban soil (not-sealed surface)<br>Generic urban soil | %| 2.37 | buildings and open area (25,026 km2) – residential space (13,125 km2) – commercial space (3,437 km2), data of 2014 (Statistisches Bundesamt 2015) |
| Total commercial space | % | 0.74 | commercial space (1,024 km2) + mining land (1,619 km2), data of 2014 (Statistisches Bundesamt 2015) |
| Total recreation area | % | 1.23 | Total (4,397 km2) incl. green area/parks (2,900 km2), data of 2014 (Statistisches Bundesamt 2015) |
| Burial ground | % | 0.11 | 377 km2, data of 2014 (Statistisches Bundesamt 2015) |
| Area of urban soil (sealed surface) | % | 9.69 | residential space (13,125 km2) + industrial space (3,437 km2) + traffic space (18,071 km2), data of 2014 (Statistisches Bundesamt 2015) |
| Area covered with fresh water | % | 2.37 | 8,477 km2, data of 2014 (Statistisches Bundesamt 2015) |
| Area of sludge treated soil | $km^2$ | 4,074 | Equal to the minimum sewage-treated area assuming 5t per ha over three years will be accepted, data of 2014 (Statistisches Bundesamt 2016) |
| Mean depth of natural soil | m | <br>0.05<br>0.1 | Possible range of the natural soil depth<br>ECB (2003)<br>Vanwalleghem, Poesen et al. (2010) |
| Mean depth agricultural soil | m | 0.2 | (ECB 2003) |
| Mean depth urban soil | m | 0.05 | (ECB 2003) |
| Soil density | kg $m^{-3}$ | 1,500 | Dry soil density (ECB 2003) |
| Water-covered surface (sea waters) | $km^2$ | 55,499 | (World Resources Institute 2010) |
| Mean depth fresh water | m | 3 | (ECB 2003) |

| | | | |
|---|---|---|---|
| Mean depth sea water | m | 10 | (Gottschalk, Lassen et al. 2015, Gottschalk, Nowack et al. 2015) |
| Sediment density | kg $m^{-3}$ | 260 | Dry sediment density (ECB 2003) |
| Coast line (sea water) | km | 3,624 | (World Resources Institute 2010) |
| Coast line (fresh water) | km | 104,208 | The total length of German rivers and streams (> 10 km) is 52,104 km (own calculation based on Wikipedia (2017).<br>This has to be multiplied by 2 as each stream/river has two banks/shores. |
| **Aquatic parameters** | | | |
| Daily water consumption per inhabitant | l/d | 121.2 | Households and small enterprises, 2013 (Statistisches Bundesamt 2016) |
| Residence time (water in a particular river from the source to the sea) | days | 10 | Most of the rivers, considering that small rivers represent inflows of larger rivers. |
| Sewage treatment plants connected to freshwater and sea water | % | Fresh-water 98.8<br><br>Sea water 1.2 | Own calculation according to Niedersächsisches Ministerium für Umwelt (2015), Ministerium für Energiewende (2015), Landesamt für Umwelt (2015), bdew (2016), BMUB (2014) |
| **Waste handling** | | | |
| Annual sewage treatment sludge production | $t_{dw}$/y | 1,796,398 | Average of 2012, 13, 14 (Statistisches Bundesamt 2016) |
| Annual volume of slag from WIPs | t/y | 5,203,000 | (Alwast and Riemann 2010, p. 35) |
| Annual volume of fly ash from WIPs | t/y | 1,040,600 | According to the ratio fly ash to slag from WIP (modal value) taken from elsewhere (Gottschalk, Lassen et al. 2015). |
| Annual volume of solid waste treated in WIP | t/y | 38,509,200 | Data for 2014 (Statistisches Bundesamt 2016) |

## 7. Supplementary on stochastic ENM life cycle based dynamic release

**Data collection**

The collection of ENM containing products and their categorization into relevant applications was conducted by means of a standard questionnaire and publicly available databases:
a. The Project on Emerging Nanotechnologies, Consumer Products Inventory
http://www.nanotechproject.org/cpi/browse/nanomaterials/
b. nanowatch.de
http://www.bund.net/nc/themen_und_projekte/nanotechnologie/nanoproduktdatenbank/produktsuche/
c. ANEC/beuc inventory of nanoproducts 2010
http://www.beuc.eu/publications/2013-00141-01-e.xls
d. The Nanodatabase
http://nanodb.dk
e. Nanowerk-Database
http://www.nanowerk.com/
g. internet-search by Google

**ENM in nature for use (USE) and end of life (EOL) material phases.**

The following material input (I) functions refer to the ENM already and persistently out there in nature or technical sinks summing up to a certain time point when material is not anymore in consumption or in release processes. The first equation refers to USE release, the second is an equivalent example for EOL modeling.

$$r_{out\_there\_use}(t) = \sum_j \frac{I(t_j) \cdot (t-j)}{m} \quad (1)$$

$$r_{out\_there\_eol}(t) = \sum_{j=m+1} I(t_{j-m}) \qquad (t \leq 2m) \quad (2)$$

---

```
Nt <- 1000
    Ot <- matrix(0,n, Nt)
      for (k in 1:Nt){
          Rt <- c(rep(0,nt))
          R[1] t <- c(input mass1 [k]t)
          …
          R[n] t <- c(input massn [k]t)

          Tt <- matrix(0,n,n)
          T [2,1] t <- -(mf1,2[k]t)
          …
          T [n,1] t <- -(mf1,n[k]t)

       O [,k] t <- solve(Tt,Rt)}
```

Nt: Number of Monte Carlo computations at time t
Ot: Matrix of the dynamic (non-stable over time) model input-output balance at time t
Rt: Vector of the dynamic material input (release) into the system occurring at different locations (boxes) at time t
Tt: Matrix reflecting the dynamic (non-stable over time) transfer coefficients at time t
mf1,2t: Mass fraction transferred from box 1 to box 2 (boxes represent locations or material states over the life cycle of a target material) at time t
nt: Number of boxes releasing or receiving a target material at time t

---

**Figure S1. Simplified conceptual Monte Carlo R approach of the dynamic stochastic ENM (engineered nanomaterial) life cycle based mass transfer**

## 8. Flow charts for 2017

Figure S2-4. describe flow chart snapshots in t/a for 2017 of a non-stable but permanently changing system for $SiO_2$-ENM (Figure S2), $CeO_2$-ENM (Figure S3), and Ag-ENM (Figure S4). The upper figures show in each case engineered nanomaterial (ENM) use based release (USE). The lower figures represent in each case the total of USE and end of life treatment (EOL) ENM release. The values given indicate only a rough trend of mean values, with limited informative explanatory power of single values taken from probability distributions. Mass balance is in principle found in such snapshots, however a bit lost because of rounding and taking together values in varying magnitudes.

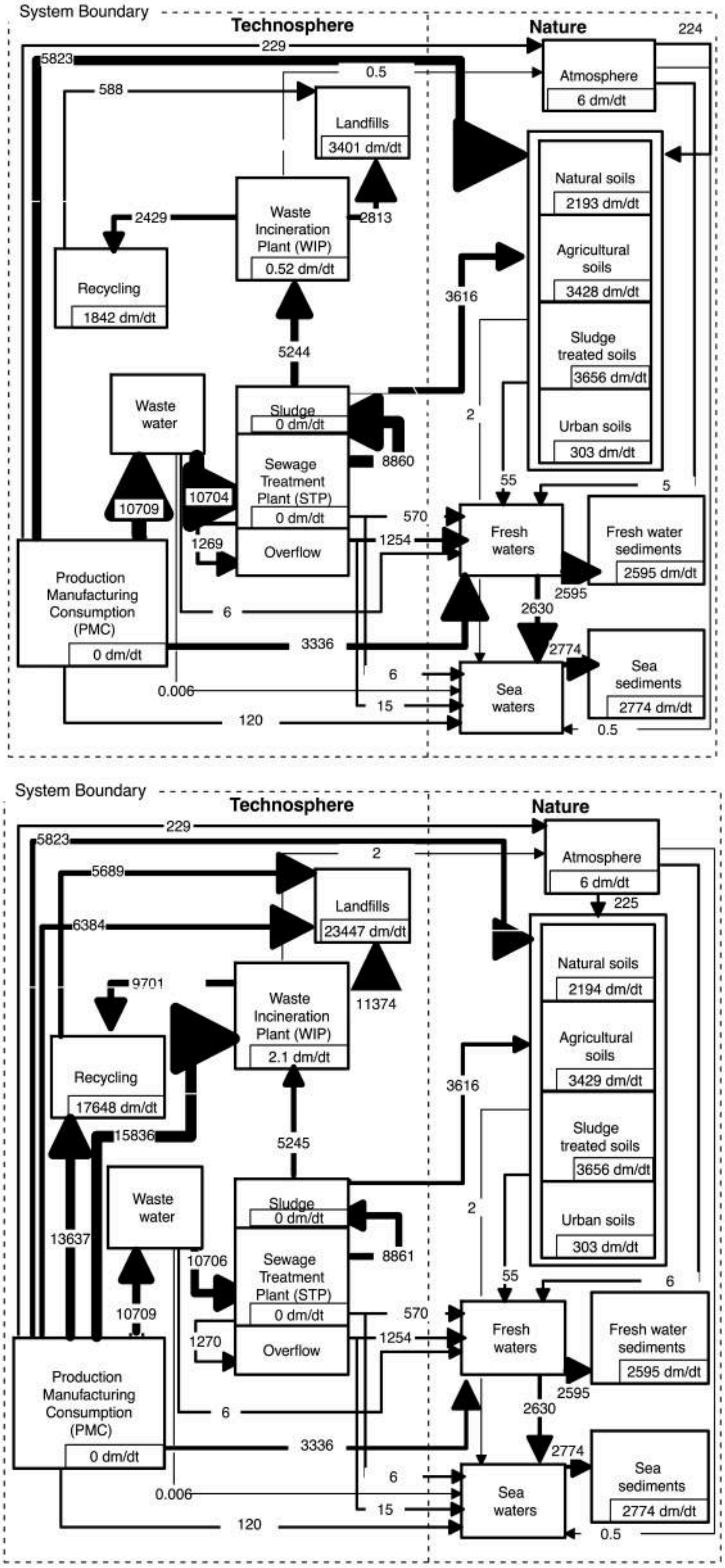


**Fig. S2. $SiO_2$-ENM**

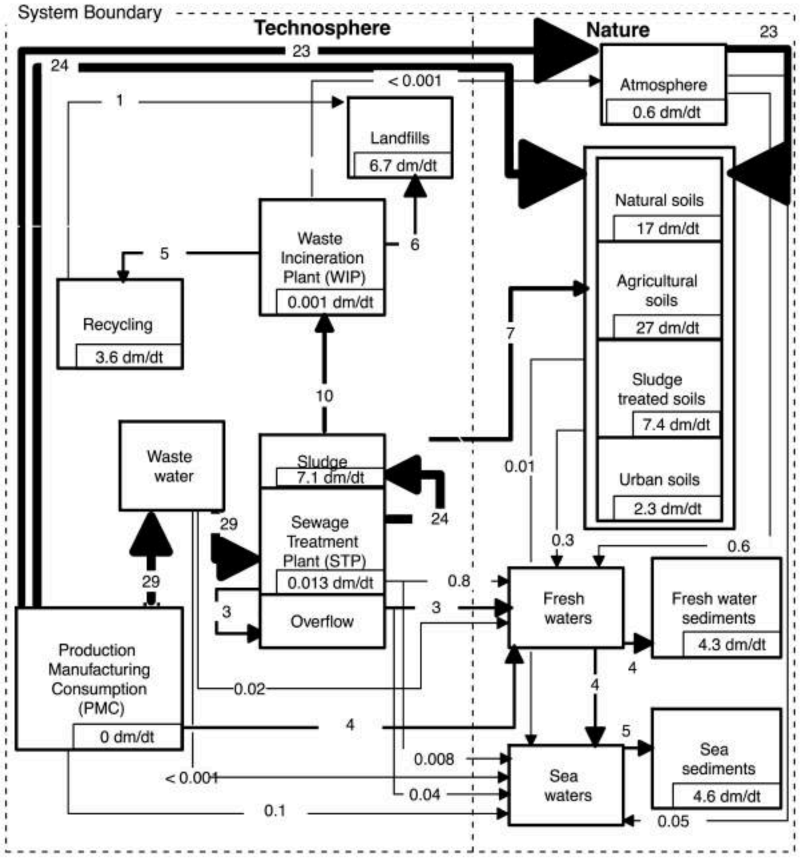


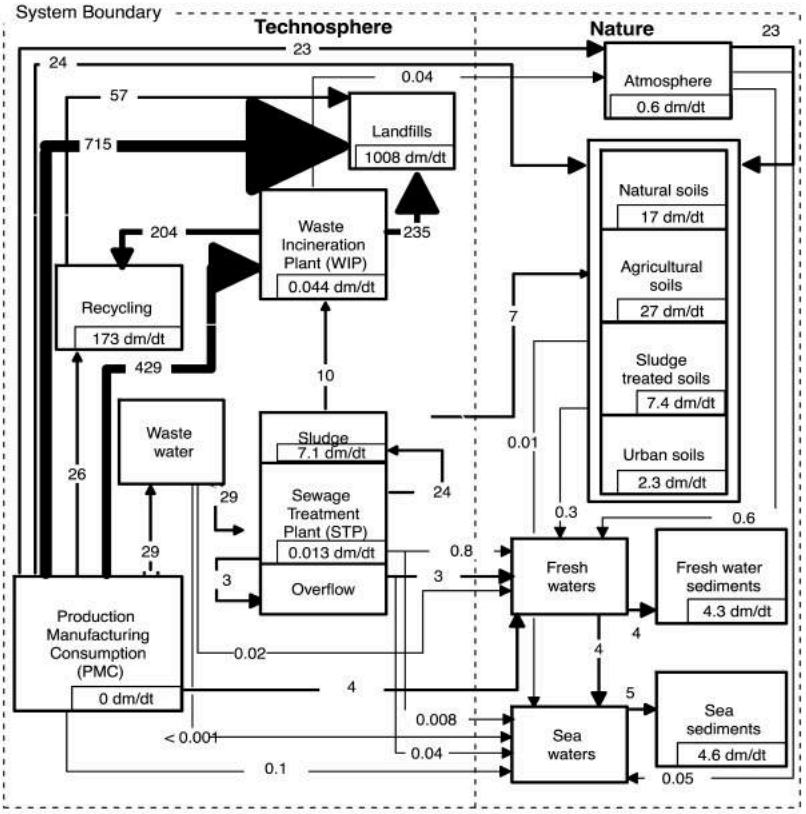


**Fig. S3. $CeO_2$-ENM**

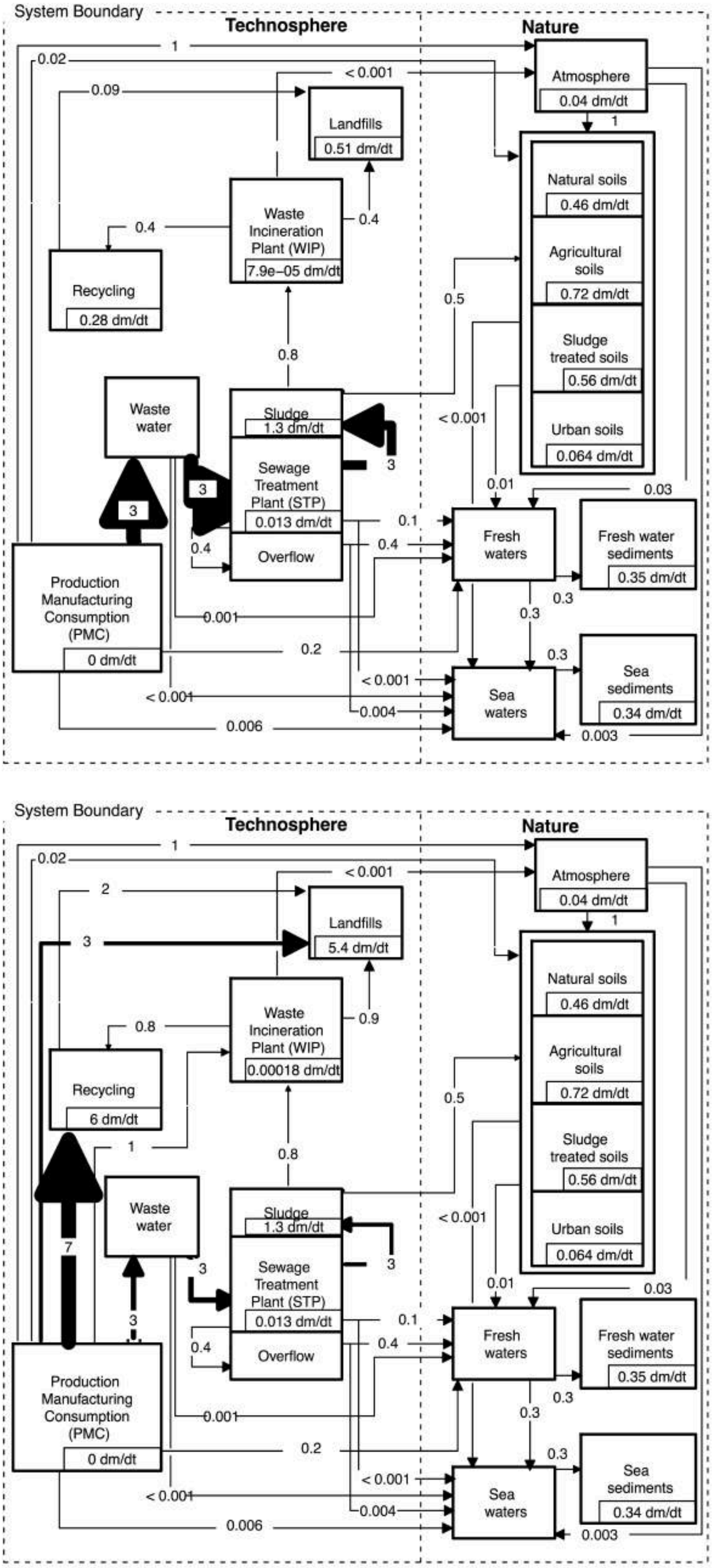
System Boundary
Technosphere
Nature
Atmosphere
0.04 dm/dt
Landfills
0.51 dm/dt
Natural soils
0.46 dm/dt
Waste Incineration Plant (WIP)
7.9e−05 dm/dt
Agricultural soils
0.72 dm/dt
Recycling
0.28 dm/dt
Sludge treated soils
0.56 dm/dt
Waste water
Sludge
1.3 dm/dt
Urban soils
0.064 dm/dt
Sewage Treatment Plant (STP)
0.013 dm/dt
Overflow
Fresh waters
Fresh water sediments
0.35 dm/dt
Production Manufacturing Consumption (PMC)
0 dm/dt
Sea waters
Sea sediments
0.34 dm/dt
1
0.02
< 0.001
0.09
0.4
0.4
0.8
0.5
3
3
3
< 0.001
0.01
0.03
0.1
0.4
0.4
0.001
0.2
0.3
0.3
0.3
< 0.001
< 0.001
0.004
0.006
0.003
System Boundary
Technosphere
Nature
Atmosphere
0.04 dm/dt
Landfills
5.4 dm/dt
Natural soils
0.46 dm/dt
Waste Incineration Plant (WIP)
0.00018 dm/dt
Agricultural soils
0.72 dm/dt
Recycling
6 dm/dt
Sludge treated soils
0.56 dm/dt
Waste water
Sludge
1.3 dm/dt
Urban soils
0.064 dm/dt
Sewage Treatment Plant (STP)
0.013 dm/dt
Overflow
Fresh waters
Fresh water sediments
0.35 dm/dt
Production Manufacturing Consumption (PMC)
0 dm/dt
Sea waters
Sea sediments
0.34 dm/dt
1
0.02
< 0.001
2
3
0.8
0.9
1
0.8
0.5
7
3
3
3
< 0.001
0.01
0.03
0.1
0.4
0.4
0.001
0.2
0.3
0.3
0.3
< 0.001
< 0.001
0.004
0.006
0.003

**Fig. S4. Ag-ENM**

## 9. Predicted environmental concentrations

Predicted environmental concentrations (PEC) for 2017, 2030 and 2050 are shown for $SiO_2$-ENM, $CeO_2$-ENM and Ag-ENM. We show on the one hand the modeled mode values, the mode values that only reflect concentrations from EOL (end of life ) releases, the modeled ranges that represent values with “some probability”. Such “some probability” simply refers to the results range of our main modeling where we did not per se force all applications running at the lowest or highest production levels and environmental release levels. Such simultaneous scenarios of “all engineered nanomaterial applications running at the same time” at their lowest and in another case at their highest production, use and release levels were modelled separately and provided the total min-max range. We have rounded the model output (3 places after the decimal point). Nevertheless, those values after the decimal point should not suggest any accuracy of individual values taken from the analysis of probability distributions.

**Table S 15: Predicted environmental concentrations for $SiO_2$-ENM (PEC)**

| $SiO_2$ - 2017 | Unit | Min | Mode | (Mode EOL) | Range with "some" probability | | Max |
|---|---|---|---|---|---|---|---|
| **Technical compartments** | | | | | | | |
| Sewage treatment effluent | ng/L | 5.418 | 44,372.013 | 6.433 | 9,038.635 | – 821,531.917 | 1,557,608.364 |
| Sewage treatment sludge | µg/kg | 185.528 | 4,955,150.406 | 668.406 | 1,746,586.371 | – 10,290,864.703 | 18,850,636.235 |
| Waste mass incinerated | µg/kg | 23.035 | 506,032.560 | 369,831.000 | 194,825.860 | – 1,154,574.800 | 2,091,707.182 |
| Bottom ash | µg/kg | 127.830 | 2,808,183.400 | 2,052,342.000 | 1,081,171.200 | – 6,407,206.600 | 11,607,727.091 |
| Fly ash | µg/kg | 213.050 | 4,680,305.800 | 3,420,570.000 | 1,801,952.000 | – 10,678,678.000 | 19,346,208.545 |
| **Natural compartments** | | | | | | | |
| Surface water (fresh water) | ng/L | 0.253 | 5,341.495 | 0.255 | 1,797.750 | – 11,634.808 | 25,551.639 |
| Sea water | ng/L | 0.000 | 0.121 | 0.000 | 0.005 | – 0.385 | 1.112 |
| Sediments (fresh water)* | µg/kg | 0.137 | 32,118.755 | 1.535 | 145.219 | – 131,134.503 | 302,822.828 |
| Sediments (fresh water)** | µg/kg | 3.938 | 920,808.569 | 38.569 | 4,627.205 | – 3,849,294.681 | 8,681,589.792 |
| Sediments (sea water)* | µg/kg | 0.073 | 5,675.395 | 0.257 | 217.263 | – 18,027.583 | 52,020.284 |
| Sediments (sea water)** | µg/kg | 1.998 | 154,524.586 | 6.086 | 6,981.061 | – 618,617.047 | 1,416,361.943 |
| Agricultural soils* | ng/kg | 2.853 | 62,823.534 | 13.394 | 25,607.468 | – 127,753.670 | 258,167.972 |
| Agricultural soils** | ng/kg | 77.140 | 1,698,930.895 | 325.895 | 945,724.128 | – 2,622,648.031 | 6,981,612.018 |
| Natural soils* | ng/kg | 7.053 | 155,331.178 | 33.118 | 63,314.453 | – 315,871.008 | 638,320.226 |
| Natural soils** | ng/kg | 190.729 | 4,200,607.774 | 805.774 | 2,338,302.830 | – 6,484,496.892 | 17,262,039.358 |
| Urban soils* | ng/kg | 11.285 | 248,529.788 | 52.988 | 101,303.117 | – 505,393.412 | 1,021,312.198 |
| Urban soils** | ng/kg | 305.166 | 6,720,971.239 | 1,289.239 | 3,741,284.128 | – 10,375,195.828 | 27,619,264.287 |

| | | | | | | | |
|---|---|---|---|---|---|---|---|
| Sludge treated soils* | ng/kg | 112.141 | 3,085,149.673 | 421.673 | 1,125,362.260 | – 6,508,828.958 | 11,603,863.665 |
| Sludge treated soils** | ng/kg | 2,845.189 | 78,275,109.732 | 9,413.732 | 32,021,292.796 | – 177,840,159.500 | 294,408,310.138 |
| Air | ng/m3 | 0.000 | 13.924 | 0.098 | 1.893 | – 51.362 | 79.426 |

* 100% degradation scenario after one year
** 100% persistent engineered nanomaterial scenario

| $SiO_2$ - 2030 | Unit | Min | Mode | (Mode EOL) | Range | | Max |
|---|---|---|---|---|---|---|---|
| **Technical compartments** | | | | | | | |
| Sewage treatment effluent | ng/L | 9.563 | 73,925.794 | 10.294 | 19,367.042 | – 1,487,498.990 | 3,167,978.537 |
| Sewage treatment sludge | µg/kg | 342.746 | 9,401,005.783 | 1,095.783 | 3,849,284.323 | – 22,309,987.030 | 41,250,888.568 |
| Waste mass incinerated | µg/kg | 39.831 | 915,519.200 | 608,755.000 | 350,194.460 | – 2,194,586.400 | 4,089,684.273 |
| Bottom ash | µg/kg | 221.040 | 5,080,537.200 | 3,378,198.000 | 1,943,358.600 | – 12,178,526.000 | 22,695,212.455 |
| Fly ash | µg/kg | 368.400 | 8,467,562.000 | 5,630,330.000 | 3,238,930.200 | – 20,297,544.000 | 37,825,355.455 |
| **Natural compartments** | | | | | | | |
| Surface water (fresh water) | ng/L | 0.330 | 9,490.603 | 0.483 | 3,658.760 | – 20,972.125 | 49,027.308 |
| Sea water | ng/L | 0.000 | 0.196 | 0.000 | 0.010 | – 0.892 | 1.706 |
| Sediments (fresh water)* | µg/kg | 0.925 | 56,577.555 | 3.115 | 513.468 | – 272,751.369 | 598,257.991 |
| Sediments (fresh water)** | µg/kg | 24.827 | 1,517,780.326 | 64.926 | 7,391.602 | – 6,460,004.300 | 16,049,194.989 |
| Sediments (sea water)* | µg/kg | 0.177 | 9,150.147 | 0.403 | 487.917 | – 41,759.996 | 79,832.407 |
| Sediments (sea water)** | µg/kg | 4.893 | 253,218.672 | 10.472 | 11,605.760 | – 1,015,489.211 | 2,209,260.350 |
| Agricultural soils* | ng/kg | 5.074 | 113,694.966 | 22.046 | 49,930.446 | – 222,188.718 | 475,688.277 |
| Agricultural soils** | ng/kg | 127.277 | 2,851,790.259 | 564.259 | 1,587,696.650 | – 4,402,171.550 | 11,931,602.943 |

| | | | | | | | |
|---|---|---|---|---|---|---|---|
| Natural soils* | ng/kg | 12.546 | 281,110.910 | 54.510 | 123,453.011 | – 549,361.735 | 1,176,139.119 |
| Natural soils** | ng/kg | 314.693 | 7,051,049.131 | 1,395.131 | 3,925,579.580 | – 10,884,369.732 | 29,500,863.984 |
| Urban soils* | ng/kg | 20.074 | 449,777.216 | 87.216 | 197,524.825 | – 878,978.535 | 1,881,822.755 |
| Urban soils** | ng/kg | 503.508 | 11,281,678.210 | 2,232.210 | 6,280,927.727 | – 17,414,991.572 | 47,201,409.996 |
| Sludge treated soils* | ng/kg | 164.996 | 4,372,365.208 | 513.208 | 1,832,880.864 | – 10,101,406.555 | 18,693,550.469 |
| Sludge treated soils** | ng/kg | 4,873.694 | 129,152,203.226 | 16,495.226 | 52,309,558.260 | – 291,015,328.100 | 552,175,564.981 |
| Air | ng/m3 | 0.001 | 28.665 | 0.162 | 4.945 | – 86.309 | 151.377 |

* 100% degradation scenario after one year

** 100% persistent engineered nanomaterial scenario

| $SiO_2$ - 2050 | Unit | Min | Mode | (Mode EOL) | Range | | Max |
|---|---|---|---|---|---|---|---|
| **Technical compartments** | | | | | | | |
| Sewage treatment effluent | ng/L | 21.002 | 200,101.418 | 25.418 | 34,493.192 | – 4,202,908.826 | 7,322,910.585 |
| Sewage treatment sludge | µg/kg | 931.499 | 23,243,033.636 | 2,959.636 | 7,543,975.563 | – 57,141,363.716 | 99,716,088.715 |
| Waste mass incinerated | µg/kg | 102.383 | 2,469,728.200 | 1,719,907.200 | 851,121.400 | – 5,340,568.000 | 10,180,727.182 |
| Bottom ash | µg/kg | 568.162 | 13,705,534.000 | 9,544,468.000 | 4,723,191.600 | – 29,637,046.000 | 56,496,804.545 |
| Fly ash | µg/kg | 946.936 | 22,842,556.000 | 15,907,448.000 | 7,871,984.000 | – 49,395,076.000 | 94,161,346.364 |
| **Natural compartments** | | | | | | | |
| Surface water (fresh water) | ng/L | 1.104 | 25,318.063 | 1.343 | 9,888.993 | – 62,582.847 | 127,018.904 |
| Sea water | ng/L | 0.000 | 0.502 | 0.000 | 0.021 | – 2.346 | 4.202 |
| Sediments (fresh water)* | µg/kg | 5.235 | 156,486.195 | 8.435 | 927.495 | – 800,476.150 | 1,258,230.328 |
| Sediments (fresh water)** | µg/kg | 122.486 | 3,661,234.438 | 158.438 | 16,109.482 | – 15,043,973.492 | 29,438,227.491 |
| Sediments (sea | µg/kg | 0.888 | 23,511.657 | 1.197 | 984.022 | – 109,788.737 | 196,631.095 |

| | | | | | | | |
|---|---|---|---|---|---|---|---|
| water)* | | | | | | | |
| Sediments (sea water)** | µg/kg | 22.088 | 584,513.641 | 25.041 | 27,029.992 | – 2,412,566.027 | 4,888,364.890 |
| Agricultural soils* | ng/kg | 13.681 | 292,423.292 | 62.292 | 89,501.510 | – 633,864.704 | 1,250,317.796 |
| Agricultural soils** | ng/kg | 327.875 | 7,008,187.894 | 1,373.894 | 3,777,272.853 | – 10,648,264.626 | 29,964,993.471 |
| Natural soils* | ng/kg | 33.826 | 723,016.418 | 154.018 | 221,292.528 | – 1,567,230.631 | 3,091,411.260 |
| Natural soils** | ng/kg | 810.672 | 17,327,746.952 | 3,396.952 | 9,339,306.118 | – 26,327,832.258 | 74,088,486.435 |
| Urban soils* | ng/kg | 54.122 | 1,156,826.228 | 246.428 | 354,067.964 | – 2,507,569.569 | 4,946,257.853 |
| Urban soils** | ng/kg | 1,297.075 | 27,724,395.122 | 5,435.122 | 14,942,888.189 | – 42,124,532.414 | 118,541,578.482 |
| Sludge treated soils* | ng/kg | 443.085 | 10,737,235.822 | 1,403.822 | 3,432,815.540 | – 28,094,476.242 | 46,386,324.347 |
| Sludge treated soils** | ng/kg | 11,064.928 | 268,135,510.000 | 34,538.000 | 104,646,444.462 | – 614,841,749.700 | 1,158,382,002.756 |
| Air | ng/m3 | 0.002 | 84.944 | 0.457 | 9.906 | – 271.117 | 431.137 |

* 100% degradation scenario after one year

** 100% persistent engineered nanomaterial scenario

**Table S 16: Predicted environmental concentrations for $CeO_2$-ENM (PEC)**

| $CeO_2$ - 2017 | Unit | Min | Mode | (From EOL) | Range with "some" probability | | | Max |
|---|---|---|---|---|---|---|---|---|
| **Technical compartments** | | | | | | | | |
| Sewage treatment effluent | ng/L | 0.035 | 199.923 | 0.260 | 20.299 | – | 888.754 | 1,867.897 |
| Sewage treatment sludge | µg/kg | 1.529 | 6,694.723 | 10.675 | 749.999 | – | 31,302.121 | 53,596.213 |
| Waste mass incinerated | µg/kg | 1.960 | 8,361.053 | 8,175.334 | 709.183 | – | 43,693.362 | 72,329.818 |
| Bottom ash | µg/kg | 10.874 | 46,399.392 | 45,368.760 | 3,935.512 | – | 242,474.472 | 401,388.944 |
| Fly ash | µg/kg | 18.123 | 77,332.320 | 75,614.600 | 6,559.187 | – | 404,124.252 | 668,981.845 |
| **Natural compartments** | | | | | | | | |
| Surface water (fresh water) | ng/L | 0.001 | 7.006 | 0.006 | 0.507 | – | 25.585 | 61.738 |
| Sea water | ng/L | 0.000 | 0.000 | 0.000 | 0.000 | – | 0.001 | 0.003 |
| Sediments (fresh water)* | µg/kg | 0.002 | 49.029 | 0.024 | 0.344 | – | 253.490 | 611.949 |
| Sediments (fresh water)** | µg/kg | 0.043 | 901.122 | 0.544 | 4.398 | – | 8,328.880 | 13,496.607 |
| Sediments (sea water)* | µg/kg | 0.000 | 5.961 | 0.004 | 0.309 | – | 43.589 | 120.261 |
| Sediments (sea water)** | µg/kg | 0.009 | 144.153 | 0.086 | 5.348 | – | 1,329.197 | 2,908.228 |
| Agricultural soils* | ng/kg | 0.099 | 377.409 | 0.296 | 43.675 | – | 1,491.873 | 2,943.373 |
| Agricultural soils** | ng/kg | 2.095 | 7,963.940 | 6.236 | 1,314.061 | – | 36,210.876 | 62,109.918 |
| Natural soils* | ng/kg | 0.246 | 933.144 | 0.732 | 107.985 | – | 3,688.656 | 7,277.494 |
| Natural soils** | ng/kg | 5.181 | 19,690.840 | 15.418 | 3,249.015 | – | 89,531.401 | 153,566.844 |
| Urban soils* | ng/kg | 0.393 | 1,493.030 | 1.171 | 172.777 | – | 5,901.852 | 11,643.990 |
| Urban soils** | ng/kg | 8.289 | 31,505.349 | 24.669 | 5,198.423 | – | 143,250.241 | 245,706.979 |
| Sludge treated soils* | ng/kg | 0.975 | 4,450.166 | 6.646 | 528.574 | – | 19,011.989 | 35,069.364 |
| Sludge treated soils** | ng/kg | 25.959 | 118,515.877 | 138.037 | 11,212.112 | – | 560,423.284 | 933,959.893 |
| Air | ng/m3 | 0.000 | 1.227 | 0.002 | 0.115 | – | 5.534 | 12.104 |

* 100% degradation scenario after one year

** 100% persistent engineered nanomaterial scenario

| $CeO_2$ - 2030 | Unit | Min | Mode | (From EOL) | Range | | Max |
|---|---|---|---|---|---|---|---|
| **Technical compartments** | | | | | | | |
| Sewage treatment effluent | ng/L | 0.052 | 347.179 | 0.419 | 31.474 | – 1,607.455 | 3,392.329 |
| Sewage treatment sludge | µg/kg | 2.702 | 13,752.341 | 17.389 | 1,470.580 | – 55,386.060 | 103,735.245 |
| Waste mass incinerated | µg/kg | 4.001 | 13,797.984 | 13,351.304 | 1,417.554 | – 79,512.093 | 151,307.962 |
| Bottom ash | µg/kg | 22.203 | 76,571.298 | 74,092.500 | 7,866.597 | – 441,249.130 | 839,673.112 |
| Fly ash | µg/kg | 37.005 | 127,618.810 | 123,487.480 | 13,110.996 | – 735,415.216 | 1,399,455.464 |
| **Natural compartments** | | | | | | | |
| Surface water (fresh water) | ng/L | 0.002 | 14.424 | 0.009 | 1.639 | – 59.925 | 99.457 |
| Sea water | ng/L | 0.000 | 0.000 | 0.000 | 0.000 | – 0.002 | 0.004 |
| Sediments (fresh water)* | µg/kg | 0.006 | 70.789 | 0.041 | 1.505 | – 556.572 | 1,047.668 |
| Sediments (fresh water)** | µg/kg | 0.137 | 1,561.652 | 0.966 | 8.530 | – 13,765.613 | 23,112.081 |
| Sediments (sea water)* | µg/kg | 0.001 | 12.176 | 0.007 | 0.390 | – 79.788 | 188.619 |
| Sediments (sea water)** | µg/kg | 0.013 | 253.163 | 0.157 | 10.076 | – 2,201.393 | 3,921.705 |
| Agricultural soils* | ng/kg | 0.181 | 531.819 | 0.484 | 97.774 | – 2,862.890 | 5,908.254 |
| Agricultural soils** | ng/kg | 4.754 | 13,940.244 | 11.436 | 2,291.012 | – 64,004.104 | 154,869.316 |
| Natural soils* | ng/kg | 0.448 | 1,314.923 | 1.196 | 241.747 | – 7,078.496 | 14,608.162 |
| Natural soils** | ng/kg | 11.753 | 34,467.255 | 28.275 | 5,664.528 | – 158,250.141 | 382,914.495 |
| Urban soils* | ng/kg | 0.717 | 2,103.877 | 1.913 | 386.795 | – 11,325.594 | 23,373.059 |
| Urban soils** | ng/kg | 18.805 | 55,147.600 | 45.240 | 9,063.244 | – 253,200.306 | 612,663.258 |
| Sludge treated soils* | ng/kg | 1.447 | 6,881.374 | 8.468 | 736.370 | – 28,461.958 | 53,922.294 |
| Sludge treated soils** | ng/kg | 43.318 | 205,965.562 | 248.962 | 19,455.773 | – 916,016.200 | 1,613,941.673 |
| Air | ng/m3 | 0.001 | 1.950 | 0.004 | 0.268 | – 12.035 | 20.343 |

* 100% degradation scenario after one year
** 100% persistent engineered nanomaterial scenario

| $CeO_2$ 2050 | Unit | Min | Mode | (From EOL) | Range | | Max |
|---|---|---|---|---|---|---|---|
| **Technical compartments** | | | | | | | |
| Sewage treatment effluent | ng/L | 0.164 | 981.829 | 1.045 | 82.602 | – 3,665.494 | 8,666.825 |
| Sewage treatment sludge | µg/kg | 7.755 | 39,165.284 | 52.564 | 3,625.771 | – 125,886.303 | 290,329.603 |
| Waste mass incinerated | µg/kg | 9.764 | 42,276.688 | 41,046.420 | 3,220.348 | – 180,092.268 | 386,716.197 |
| Bottom ash | µg/kg | 54.186 | 234,608.976 | 227,781.800 | 17,870.994 | – 999,400.180 | 2,146,051.527 |
| Fly ash | µg/kg | 90.310 | 391,014.826 | 379,636.200 | 29,784.983 | – 1,665,666.960 | 3,576,751.636 |
| **Natural compartments** | | | | | | | |
| Surface water (fresh water) | ng/L | 0.006 | 46.749 | 0.022 | 4.489 | – 148.929 | 287.016 |
| Sea water | ng/L | 0.000 | 0.001 | 0.000 | 0.000 | – 0.006 | 0.012 |
| Sediments (fresh water)* | µg/kg | 0.027 | 179.362 | 0.121 | 2.354 | – 1,844.202 | 2,910.264 |
| Sediments (fresh water)** | µg/kg | 0.614 | 4,068.649 | 2.469 | 24.038 | – 34,443.140 | 66,016.546 |
| Sediments (sea water)* | µg/kg | 0.001 | 28.869 | 0.016 | 2.428 | – 298.167 | 544.909 |
| Sediments (sea water)** | µg/kg | 0.016 | 633.588 | 0.404 | 26.050 | – 5,304.063 | 11,959.069 |
| Agricultural soils* | ng/kg | 0.478 | 1,462.069 | 1.487 | 275.351 | – 7,196.176 | 15,825.285 |
| Agricultural soils** | ng/kg | 11.752 | 35,944.090 | 28.730 | 5,656.028 | – 158,504.668 | 389,055.029 |
| Natural soils* | ng/kg | 1.182 | 3,614.968 | 3.676 | 680.806 | – 17,792.547 | 39,128.006 |
| Natural soils** | ng/kg | 29.057 | 88,871.775 | 71.035 | 13,984.527 | – 391,902.705 | 961,938.176 |
| Urban soils* | ng/kg | 1.891 | 5,783.947 | 5.881 | 1,089.290 | – 28,468.071 | 62,604.837 |
| Urban soils** | ng/kg | 46.491 | 142,194.836 | 113.656 | 22,375.241 | – 627,044.408 | 1,539,102.029 |
| Sludge treated soils* | ng/kg | 4.284 | 19,882.376 | 25.520 | 2,069.125 | – 65,371.634 | 153,514.452 |
| Sludge treated soils** | ng/kg | 95.198 | 441,784.111 | 543.711 | 45,444.158 | – 1,884,668.970 | 3,411,073.507 |
| Air | ng/m3 | 0.001 | 4.751 | 0.011 | 0.596 | – 30.930 | 51.519 |

* 100% degradation scenario after one year
** 100% persistent engineered nanomaterial scenario

Table S 17: Predicted environmental concentrations for Ag-ENM (PEC)

| Ag - 2017 | Unit | Min | Mode | (From EOL) | Range with "some" probability | | | Max |
|---|---|---|---|---|---|---|---|---|
| **Technical compartments** | | | | | | | | |
| Sewage treatment effluent | ng/L | 0.064 | 18.890 | 0.001 | 1.244 | – | 103.788 | 151.245 |
| Sewage treatment sludge | µg/kg | 1.905 | 414.799 | 0.018 | 32.423 | – | 2,730.256 | 3,934.479 |
| Waste mass incinerated | µg/kg | 0.164 | 32.145 | 20.729 | 5.025 | – | 141.886 | 353.763 |
| Bottom ash | µg/kg | 0.911 | 178.386 | 115.036 | 27.886 | – | 787.385 | 1,963.171 |
| Fly ash | µg/kg | 1.518 | 297.310 | 191.726 | 46.477 | – | 1,312.308 | 3,271.951 |
| **Natural compartments** | | | | | | | | |
| Surface water (fresh water) | ng/L | 0.001 | 0.382 | 0.000 | 0.035 | – | 2.789 | 4.171 |
| Sea water | ng/L | 0.000 | 0.000 | 0.000 | 0.000 | – | 0.000 | 0.000 |
| Sediments (fresh water)* | µg/kg | 0.000 | 1.854 | 0.000 | 0.025 | – | 33.671 | 47.016 |
| Sediments (fresh water)** | µg/kg | 0.004 | 29.554 | 0.002 | 0.193 | – | 470.645 | 749.355 |
| Sediments (sea water)* | µg/kg | 0.000 | 0.328 | 0.000 | 0.009 | – | 4.189 | 6.794 |
| Sediments (sea water)** | µg/kg | 0.008 | 5.084 | 0.000 | 0.135 | – | 76.816 | 105.216 |
| Agricultural soils* | ng/kg | 0.026 | 7.759 | 0.001 | 0.236 | – | 67.727 | 80.299 |
| Agricultural soils** | ng/kg | 0.501 | 147.132 | 0.016 | 9.399 | – | 792.232 | 1,522.662 |
| Natural soils* | ng/kg | 0.065 | 19.184 | 0.002 | 0.584 | – | 167.456 | 198.539 |
| Natural soils** | ng/kg | 1.240 | 363.785 | 0.041 | 23.239 | – | 1,958.795 | 3,764.782 |
| Urban soils* | ng/kg | 0.105 | 30.695 | 0.003 | 0.934 | – | 267.929 | 317.662 |
| Urban soils** | ng/kg | 1.983 | 582.056 | 0.065 | 37.183 | – | 3,134.071 | 6,023.649 |
| Sludge treated soils* | ng/kg | 1.134 | 257.184 | 0.011 | 20.085 | – | 1,661.164 | 2,294.046 |
| Sludge treated soils** | ng/kg | 22.659 | 5,141.247 | 0.249 | 464.145 | – | 24,995.354 | 45,859.264 |
| Air | ng/m3 | 0.000 | 0.055 | 0.000 | 0.001 | – | 0.495 | 0.581 |

* 100% degradation scenario after one year

** 100% persistent engineered nanomaterial scenario

| Ag - 2030 | Unit | Min | Mode | (From EOL) | Range | | | Max |
|---|---|---|---|---|---|---|---|---|
| **Technical compartments** | | | | | | | | |
| Sewage treatment effluent | ng/L | 0.101 | 30.608 | 0.001 | 2.120 | – | 211.239 | 305.498 |
| Sewage treatment sludge | µg/kg | 3.142 | 747.196 | 0.035 | 62.254 | – | 4,553.974 | 7,166.513 |
| Waste mass incinerated | µg/kg | 0.255 | 61.123 | 36.995 | 7.983 | – | 254.744 | 699.285 |
| Bottom ash | µg/kg | 1.415 | 339.199 | 205.299 | 44.298 | – | 1,413.687 | 3,880.629 |
| Fly ash | µg/kg | 2.358 | 565.331 | 342.165 | 73.830 | – | 2,356.145 | 6,467.714 |
| **Natural compartments** | | | | | | | | |
| Surface water (fresh water) | ng/L | 0.003 | 0.889 | 0.000 | 0.063 | – | 6.258 | 8.598 |
| Sea water | ng/L | 0.000 | 0.000 | 0.000 | 0.000 | – | 0.000 | 0.000 |
| Sediments (fresh water)* | µg/kg | 0.005 | 3.808 | 0.000 | 0.043 | – | 50.715 | 55.878 |
| Sediments (fresh water)** | µg/kg | 0.076 | 62.131 | 0.003 | 0.391 | – | 1,018.228 | 1,048.561 |
| Sediments (sea water)* | µg/kg | 0.004 | 0.559 | 0.000 | 0.011 | – | 12.257 | 14.058 |
| Sediments (sea water)** | µg/kg | 0.081 | 10.104 | 0.000 | 0.305 | – | 173.178 | 253.919 |
| Agricultural soils* | ng/kg | 0.042 | 14.241 | 0.001 | 0.526 | – | 100.645 | 146.557 |
| Agricultural soils** | ng/kg | 0.857 | 293.118 | 0.029 | 18.544 | – | 1,735.721 | 3,016.624 |
| Natural soils* | ng/kg | 0.103 | 35.210 | 0.003 | 1.301 | – | 248.845 | 362.361 |
| Natural soils** | ng/kg | 2.118 | 724.733 | 0.072 | 45.851 | – | 4,291.571 | 7,458.604 |
| Urban soils* | ng/kg | 0.165 | 56.336 | 0.005 | 2.082 | – | 398.152 | 579.778 |
| Urban soils** | ng/kg | 3.388 | 1,159.573 | 0.115 | 73.362 | – | 6,866.512 | 11,933.760 |
| Sludge treated soils* | ng/kg | 1.508 | 350.970 | 0.016 | 27.211 | – | 2,122.734 | 3,488.886 |
| Sludge treated soils** | ng/kg | 45.974 | 10,701.852 | 0.444 | 940.514 | – | 53,296.448 | 106,383.765 |
| Air | ng/m3 | 0.000 | 0.099 | 0.000 | 0.002 | – | 0.734 | 1.060 |

* 100% degradation scenario after one year

** 100% persistent engineered nanomaterial scenario

| Ag - 2050 | Unit | Min | Mode | (From EOL) | Range | | | Max |
|---|---|---|---|---|---|---|---|---|
| **Technical compartments** | | | | | | | | |
| Sewage treatment effluent | ng/L | 0.329 | 76.391 | 0.003 | 6.496 | – | 472.948 | 726.097 |
| Sewage treatment sludge | µg/kg | 9.471 | 2,228.588 | 0.076 | 187.964 | – | 11,669.229 | 18,375.484 |
| Waste mass incinerated | µg/kg | 0.785 | 155.705 | 83.841 | 25.546 | – | 639.388 | 1,619.530 |
| Bottom ash | µg/kg | 4.354 | 864.060 | 465.262 | 141.765 | – | 3,548.183 | 8,987.436 |
| Fly ash | µg/kg | 7.256 | 1,440.100 | 775.436 | 236.274 | – | 5,913.638 | 14,979.060 |
| **Natural compartments** | | | | | | | | |
| Surface water (fresh water) | ng/L | 0.007 | 2.199 | 0.000 | 0.134 | – | 12.896 | 21.815 |
| Sea water | ng/L | 0.000 | 0.000 | 0.000 | 0.000 | – | 0.000 | 0.001 |
| Sediments (fresh water)* | µg/kg | 0.024 | 8.053 | 0.000 | 0.118 | – | 160.683 | 178.219 |
| Sediments (fresh water)** | µg/kg | 0.547 | 180.477 | 0.008 | 1.002 | – | 2,933.342 | 3,994.223 |
| Sediments (sea water)* | µg/kg | 0.007 | 1.254 | 0.000 | 0.019 | – | 17.437 | 35.323 |
| Sediments (sea water)** | µg/kg | 0.155 | 28.902 | 0.001 | 0.852 | – | 471.308 | 814.127 |
| Agricultural soils* | ng/kg | 0.134 | 39.628 | 0.003 | 2.223 | – | 219.630 | 345.695 |
| Agricultural soils** | ng/kg | 3.129 | 925.079 | 0.071 | 53.439 | – | 4,756.104 | 8,069.867 |
| Natural soils* | ng/kg | 0.331 | 97.981 | 0.008 | 5.497 | – | 543.034 | 854.731 |
| Natural soils** | ng/kg | 7.737 | 2,287.257 | 0.175 | 132.129 | – | 11,759.465 | 19,952.735 |
| Urban soils* | ng/kg | 0.530 | 156.770 | 0.012 | 8.795 | – | 868.855 | 1,367.569 |
| Urban soils** | ng/kg | 12.379 | 3,659.612 | 0.280 | 211.406 | – | 18,815.145 | 31,924.383 |
| Sludge treated soils* | ng/kg | 4.704 | 1,079.914 | 0.039 | 88.785 | – | 5,249.632 | 8,900.106 |
| Sludge treated soils** | ng/kg | 114.366 | 26,253.140 | 0.960 | 2,301.041 | – | 128,073.886 | 216,365.062 |
| Air | ng/m3 | 0.001 | 0.282 | 0.000 | 0.005 | – | 1.608 | 2.498 |

* 100% degradation scenario after one year

** 100% persistent engineered nanomaterial scenario

## 10. Literature


Alwast, H., Riemann, A., 2010. Verbesserung der umweltrelevanten Qualitäten von Schlacken aus Abfallverbrennungsanlagen. Umweltbundesamt. Fachgebiet III 2.4 Abfalltechnik, Abfalltechniktransfer

Anastasio, C., Martin, S.T., 2001. Atmospheric Nanoparticles. Banfield, J.F.Navrotsky, A. Reviews in Mineralogy and Geochemistry. 44, Washington, D.C.

BAH, 2015. Der Arzneimittelmarkt in Deutschland 2014Bonn.

Barton, L.E., Auffan, M., Bertrand, M., Barakat, M., Santaella, C., Masion, A., Borschneck, D., Olivi, L., Roche, N., Wiesner, M.R., Bottero, J.Y., 2014. Environmental Science & Technology 48, 7289-7296.

bdew, 2016. Abwasserdaten Deutschland - Strukturdaten der AbwasserentsorgungBonn.

Bleiwas, D.I., 2013. Potential for recovery of cerium contained in automotive catalytic converters: U.S. Geological Survey Open-File Report 2013–1037Virginia.

BMEL, 2015. Statistisches Jahrbuch über Ernährung, Landwirtschaft und Forsten der Bundesrepublik Deutschland. GmbH, L.Münster-Hiltrup.

BMUB, 2014. Wasserwirtschaft in Deutschland, Teil 1 – Grundlagen

Burkhardt, M., Englert, A., Iten, R., Schärer, S., 2011. Entsorgung nanosilberhaltiger Abfälle in der Textilindustrie - Massenflüsse und Behandlungsverfahren, HSR Hochschule für Technik. Rapperswil, Schweiz.

Caballero-Guzman, A., Sun, T., Nowack, B., 2015. Flows of engineered nanomaterials through the recycling process in Switzerland. Waste Management 36, 33-43.

Den, W., Huang, C., 2005. Electrocoagulation for removal of silica nano-particles from chemical–mechanical-planarization wastewater. Colloids and Surfaces A: Physicochemical and Engineering Aspects 254 (1–3), 81-89.

Den, W., Huang, C., 2006. Electrocoagulation of Silica Nanoparticles in Wafer Polishing Wastewater by a Multichannel Flow Reactor: A Kinetic Study. Journal of Environmental Engineering 132 (12), 1651-1658.

EC, 2012. Types and uses of nanomaterials, including safety aspects, European Commission. Brussels.

ECB, 2003. Technical Guidance Document on Risk Assessment, European Chemicals Bureau. Institute for Health and Consumer Protection, European Commission. Dublin.

ECHA, 2016. Guidance on information requirements and Chemical Safety Assessment, Chapter R.16: Environmental exposure assessmentHelsinki, Finland.

Genesis, 2016. GENESIS Online Datenbank.

Gomez-Rivera, F., Field, J.A., Brown, D., Sierra-Alvarez, R., 2012. Fate of cerium dioxide (CeO2) nanoparticles in municipal wastewater during activated sludge treatment. Bioresource Technology 108, 300-304.

Goonan, T.G., 2011. Rare Earth Elements - End Use and Recyclability, U.S. Department of the Interior, U.S. Geological Survey.

Gottschalk, F., Lassen, C., Kjoelholt , J., Christensen, F., Nowack, B., 2015a. Modeling Flows and Concentrations of Nine Engineered Nanomaterials in the Danish Environment. Int. J. Environ. Res. Public Health 12 (5), 5581-5602.

Gottschalk, F., Nowack, B., Lassen, C., Kjølholt, J., Christensen, F., 2015b. Nanomaterials in the danish environment. Modelling exposure of the danish environment to selected nanomaterials, Environmental project no. 1639, 2015 from the danish environmental protection agency. 143 pp. Http://www2.Mst.Dk/udgiv/publications/2015/01/978-87-93283-60-2.Pdf.

Gottschalk, F., Sonderer, T., Scholz, R.W., Nowack, B., 2009. Modeled environmental concentrations of engineered nanomaterials (TiO2, ZnO, Ag, CNT, fullerenes) for different regions. Environmental Science and Technololgy 43, 9216-9222.

Grass, R.N., Schälchli, J., Paunescu, D., Soellner, J.O.B., Kaegi, R., Stark, W.J., 2014. Tracking Trace Amounts of Submicrometer Silica Particles in Wastewaters and Activated Sludge Using Silica-Encapsulated DNA Barcodes. Environmental Science & Technology Letters 1 (12), 484-489.

Hedberg J., Baresel C., O, W.I., 2014. Transport and fate of silver as polymer-stabilised nanoparticles and ions in a pilot wastewater treatment plant, followed by sludge digestion and disposal of sludge/soil mixtures: a case study. J. Environ. Sci. Heal. A 49, 1416-1424.

Helmers, E., 1997. Platinum emission rate of automobiles with catalytic converters: Comparison and assessment of results from various approaches. Environ Sci Pollut Res Int 4 (2), 99-103.
Huang, C., Jiang, W., Chen, C., 2004. Nano silica removal from IC wastewater by pre-coagulation and microfiltration. Water Sci Technol 50 (12), 133-8.
IKW, 2015. Bericht Nachhaltigkeit in der Wasch-, Pflege- und Reinigungsmittelbranche in Deutschland 2013-2014Frankfurt am Main.
JACC, 2006. Synthetic Amorphous SilicaBrussels, Belgium.
Jarvie, H.P., Al-Obaidi, H., King, S.M., Bowes, M.J., Lawrence, M.J., Drake, A.F., Green, M.A., Dobson, P.J., 2009. Fate of Silica Nanoparticles in Simulated Primary Wastewater Treatment. Environmental Science & Technology 43 (22), 8622-8628.
Johnson, A.C., Park, B., 2012. Predicting contamination by the fuel additive cerium oxide engineered nanoparticles within the United Kingdom and the associated risks. Environ Toxicol Chem 31 (11), 2582-7.
Kaegi, R., Sinnet, B., Zuleeg, S., Hagendorfer, H., Mueller, E., Vonbank, R., Boller, M., Burkhardt, M., 2010. Release of silver nanoparticles from outdoor facades. Environmental Pollution 158 (9), 2900-2905.
Kaegi, R., Voegelin, A., Sinnet, B., Zuleeg, S., Hagendorfer, H., Burkhardt, M., Siegrist, H., 2011. Behavior of Metallic Silver Nanoparticles in a Pilot Wastewater Treatment Plant. Environmental Science & Technology 45 (9), 3902-3908.
Kaegi, R., Voegelina, A., Ort, C., Sinnet, B., Thalmann, B., Krismer, J., Hagendorfer, H., Elumelu, M., Mueller, E., 2013. Fate and transformation of silver nanoparticles in urban wastewater systems. Water Research 47 (12), 3866-3877.
Kannan, N., White, S.M., Whelan, M.J., 2007. Predicting diffuse-source transfers of surfactants to surface waters using SWAT. Chemosphere 66 (7), 1336-1345.
Keller, A.A., McFerran, S., Lazareva, A., Suh, S., 2013. Global life cycle releases of engineered nanomaterials. Journal of Nanoparticle Research 15 (6).
Landesamt für Umwelt, N.u.G.M.-V., 2015. Kommunale Abwasserbeseitigung in Mecklenburg-Vorpommern - Lagebericht 2015Güstrow.
Lazareva, A., Keller, A.A., 2014. Estimating Potential Life Cycle Releases of Engineered Nanomaterials from Wastewater Treatment Plants. Acs Sustainable Chemistry & Engineering 2 (7), 1656-1665.
Li, L., Hartmann, G., Doblinger, M., Schuster, M., 2013. Quantification of nanoscale silver particles removal and release from municipal wastewater treatment plants in Germany. Environ. Sci. Technol. (47), 7317-7323.
Limbach, L.K., Bereiter, R., Müller, E., Krebs, R., Gälli, R., Stark, W.J., 2008. Removal of oxide nanoparticles in a model wastewater treatment plant: Influence of agglomeration and surfactants on clearing efficiency. Environmental Science & Technology 42 (15), 5828-5833.
Liu, Y., Tourbin, M., Lachaize, S., Guiraud, P., 2013. Silica nanoparticles separation from water: aggregation by cetyltrimethylammonium bromide (CTAB). Chemosphere 92 (6), 681-7.
Lombi, E., Donner, E., Taheri, S., Tavakkoli, E., Jamting, A.K., McClure, S., Naidu, R., Miller, B.W., Scheckel, K.G., Vasilev, K., 2013. Transformation of four silver/silver chloride nanoparticles during anaerobic treatment of wastewater and post-processing of sewage sludge. Environ Pollut 176, 193-7.
Ma, R., Levard, C., Judy, J.D., Unrine, J.M., Durenkamp, M., Martin, B., Jefferson, B., Lowry, G.V., 2014. Fate of Zinc Oxide and Silver Nanoparticles in a Pilot Wastewater Treatment Plant and in Processed Biosolids. Environmental Science & Technology 48 (1), 104-112.
Mackevica, A., Olsson, M.E., Hansen, S.F., 2016. Silver nanoparticle release from commercially available plastic food containers into food simulants. Journal of Nanoparticle Research 18 (1), 5.
Meo, 2014. Stoffliche und energetische Nutzung von nachwachsenden Rohstoffen, Oleochemie – Wasch- und KörperpflegemittelBerlin.
Ministerium für Energiewende, L., Umwelt und ländliche Räume des Landes Schleswig-Holstein, 2015. Beseitigung von kommunalen Abwässern in Schleswig-Holstein, Lagebericht 2014
Niedersächsisches Ministerium für Umwelt, E.u.K., 2015. Die Beseitigung kommunaler Abwässer in Niedersachsen - Lagebericht 2015

OICA, 2015. Worldwide automobile production from 2000 to 2014 (in million vehicles). OICA, OICA.
Pan, J.R., Huang, C., Jiang, W., Chen, C., 2005. Treatment of wastewater containing nano-scale silica particles by dead-end microfiltration: evaluation of pretreatment methods. Desalination 179 (1), 31-40.
Piccinno, F., Gottschalk, F., Seeger, S., Nowack, B., 2012. Industrial Production Quantities and Uses of Ten Engineered Nanomaterials in Europe and the World. Journal of Nanoparticle Research 14:1109.
Praetorius, A., Gottschalk, F., Scheringer, M., Sani-Kast, N., Nowack, B., Hungerbuehler, K., under review. Modelling the fate of titanium dioxide, silver and zinc oxide nanoparticles in Swiss rivers at high spatial resolution†. Environmental Science: Processes & Impacts.
Praetorius, A., Scheringer, M., Hungerbuehler, K., 2012. Development of environmental fate models for engineered nanoparticles - a case study of TiO2 nanoparticles in the Rhine River. Environ Sci Technol. 46 (12), 6705-6713.
Reed, K., Cormack, A., Kulkarni, A., Mayton, M., Sayle, D., Klaessig, F., Stadler, B., 2014. Exploring the properties and applications of nanoceria: is there still plenty of room at the bottom? Environmental Science: Nano 1 (5), 390-405.
Reidy, B., Haase, A., Luch, A., Dawson, K., Lynch, I., 2013. Mechanisms of Silver Nanoparticle Release, Transformation and Toxicity: A Critical Review of Current Knowledge and Recommendations for Future Studies and Applications. Materials 6 (6), 2295.
Ricardo Energy & Environment, 2016. Support for 3rd regulatory review on nanomaterials: Interim/background Report. WORKSHOP WORKING MATERIAL Report for European Commission DG Environment
ENV.A.3/ETU/2015/0030.
Schmitt, T.G., Knerr, H., Gretzschel, O., Kolisch, G., Taudien, Y., 2016. Studie zur Relevanz, Möglichkeiten und Kosten einer Elimination von Mikroschadstoffen auf kommunalen Kläranlagen in Rheinland-Pfalz- aufgezeigt am Beispiel der Nahe - Mikro_N -. Studie im Aufgtrag des Ministeriums fu□r Umwelt, Landwirtschaft, Ernährung, Weinbau und Forsten (MULEWF) des Landes Rheinland-Pfalz, Deutschland.
Schüler, D., Buchert, M., Liu, R., Dittrich, S., Merz, C., 2011. Study on Rare Earths and Their RecyclingDarmstadt.
STATISTA, 2014. Verwendung von Kunststoff in Deutschland nach Einsatzgebieten
Statistisches Bundesamt, 2015. DESTATIS Fachserie 3, Reihe 5.1
Statistisches Bundesamt, 2016a. Ergebnisse der Bevölkerungsfortschreibung auf Grundlage des Zensus 2011
Statistisches Bundesamt, 2016b. Inländische Verwendung von Gütern (Binnennachfrage) in Milliarden Euro 2014.
Statistisches Bundesamt, 2016c. Öffentliche Abwasserbeseitigung 2013, Statistisches Bundesamt. Wiesbaden.
Statistisches Bundesamt, 2016d. Öffentliche Wasserversorgung: Anschlussgrad, Wasserabgabe an Letztverbraucher (2013)Düsseldorf.
Statistisches Bundesamt, 2016e. Trockenmasse direkt entsorgter Klärschlamm, Jahressumme [t] (2014).
Statistisches Bundesamt, 2016f. Umwelt – Abfallentsorgung, Statistisches Bundesamt. Wiesbaden.
Sun, T.Y., Bornhöft, N.A., Hungerbühler, K., Nowack, B., 2016. Dynamic Probabilistic Modeling of Environmental Emissions of Engineered Nanomaterials. Environ. Sci. Technol. 50, 4701-4711.
Sun, T.Y., Conroy, G., Donner, E., Hungerbuehler, K., Lombi, E., Nowack, B., 2015. Probabilistic modelling of engineered nanomaterial emissions to the environment: a spatio-temporal approach. Environmental Science-Nano 2 (4), 340-351.
Sun, T.Y., Gottschalk, F., Hungerbühler, K., Nowack, B., 2014. Comprehensive probabilistic modelling of environmental emissions of engineered nanomaterials. Environmental Pollution 185 (x), 69-76.
Svehla, J., Krutzler, T., Schindler, I., 2012. Stand der Technik in österreichischen GiessereienWien.
Vanwalleghem, T., Poesen, J., McBratney, A., Deckers, J., 2010. Spatial variability of soil horizon depth in natural loess-derived soils. Geoderma 157 (1-2), 37-45.
VdL, 2016. Kennzahlen zur mittelständischen Lack- und Druckfarbenindustrie in Deutschland

Walser, T., Gottschalk, F., 2014. Stochastic fate analysis of engineered nanoparticles in incineration plants. Journal of Cleaner Production. 80 (Available online), 241-251.
Walser, T., Hellweg, S., Juraske, R., Luechinger, N.A., Wang, J., Fierz, M., 2012a. Exposure to engineered nanoparticles: Model and measurements for accident situations in laboratories. Science of the Total Environment 420, 119-126.
Walser, T., Limbach, L.K., Brogioli, R., Erismann, E., Flamigni, L., Hattendorf, B., Juchli, M., Krumeich, F., Ludwig, C., Prikopsky, K., Rossier, M., Saner, D., Sigg, A., Hellweg, S., Guenther, D., Stark, W.J., 2012b. Persistence of engineered nanoparticles in a municipal solid-waste incineration plant. Nature Nanotechnology 7 (8), 520-524.
Wang, Y., Kalinina, A., Sun, T., Nowack, B., 2016a. Probabilistic modeling of the flows and environmental risks of nano-silica. Sci Total Environ 545-546, 67-76.
Wang, Y., Kalinina, A., Sun, T.Y., Nowack, B., 2016b. Probabilistic modeling of the flows and environmental risks of nano-silica. Science of the Total Environment 545-546, 67-76.
Wasukan, N., Srisung, S., Kulthong, K., Boonrungsiman, S., Maniratanachote, R., 2015. Determination of silver in personal care nanoproducts and effects on dermal exposure. Journal of Nanoparticle Research 17 (11), 425.
Wigger, H., Hackmann, S., Zimmermann, T., Koser, J., Thoming, J., von Gleich, A., 2015. Influences of use activities and waste management on environmental releases of engineered nanomaterials. Sci Total Environ 535, 160-71.
Wikipedia, 2017. Liste von Flüssen in Deutschland.
World Resources Institute, 2010b. Coastal and Marine Ecosystems, Marine Jurisdictions: Continental shelf area, Germany.
Worldbank, 2016. GDP at market prices (current US$) 2014
